# A potential of serum anti-C1P IgG antibodies as biomarkers in differential diagnosis of relapsing-remitting multiple sclerosis

**DOI:** 10.1038/s41598-026-43823-y

**Published:** 2026-03-19

**Authors:** Justyna Chojdak-Lukasiewicz, Anna Jakubiak-Augustyn, Zdzislaw M. Szulc, Jerzy Gubernator, Pawel Blazej, Anna Pokryszko-Dragan, Slawomir Budrewicz, Maria Podbielska

**Affiliations:** 1 Department of Neurosurgery and Neurology, Wroclaw Medical University, Borowska 213, Wroclaw, 50-556 Poland; 2 Department of Lipids and Liposomes, University of Wroclaw, F. Joliot-Curie 14a, Wroclaw, 50-383 Poland; 3 Department of Bioinformatics and Genomics, University of Wroclaw, F. Joliot-Curie 14a, Wroclaw, 50-383 Poland; 4 Department of Biochemistry & Molecular Biology, Medical University of South Carolina, 173 Ashley Avenue, Charleston, SC 29425-2503 USA; 5 Laboratory of Microbiome Immunobiology, Ludwik Hirszfeld Institute of Immunology & Experimental Therapy, Polish Academy of Sciences, Rudolfa Weigla 12, Wroclaw, 53-114 Poland

**Keywords:** Biomarkers, Ceramide, C1P, Lipid antigens, Multiple sclerosis, Sphingolipids, Biomarkers, Diseases, Immunology, Neurology, Neuroscience

## Abstract

There is a growing interest in the role of sphingolipids in the background of multiple sclerosis (MS). The goal of this study was to evaluate the serum levels of antibodies against ceramide-1-phosphate (C1P) subclasses and their relationships with clinical status in MS. The study groups comprised 39 patients with relapsing-remitting MS (RRMS), 26 patients with other neurological diseases (OND) and 12 healthy subjects (HS). Anti-C1P IgG levels in serum were determined using ELISA test. Levels of anti-C18:0-C1P and anti-C24:1-C1P IgG were significantly increased (*p* = 0.003; *p* < 0.0001, respectively) in RRMS compared to HS, while anti-C16:0-C1P and anti-C24:0-C1P IgG – significantly lower *p* < 0.0001 in RRMS compared to OND, with large effect size (*r* ≥ 0.5) in all above cases. In both settings the acceptable discriminatory performance for RRMS subtype from HS (AUC = 78.1%, 95% CI 66.1–90.4 and AUC = 76.9%, 95% CI 60.3–93.6, respectively) as well as from OND (AUC = 79.8%, 95% CI 68.2–91.4 and AUC = 94.2%, 95% CI 88.6–99.8, accordingly) by receiver operating curve (ROC) was shown. Validation of ROC by cluster analysis confirmed the ability of these anti-C1P IgG panels to discriminate between the study groups. No relationships were found between levels of antibodies in the anti-C1P IgG panel in RRMS group and disease duration, degree of disability or Link index. These findings highlight the relevant role of C1P as a target and/or mediator of autoimmune response in MS and potential value of anti-C1P antibodies as biomarkers in differential diagnosis of this disease.

**Supplementary Information:**

The online version contains supplementary material available at 10.1038/s41598-026-43823-y.

## Introduction

Multiple sclerosis (MS) is a chronic polyphasic disease of the central nervous system (CNS) which causes multifocal damage to the brain and the spinal cord and results in multiple aspects of disability. The complex background of MS involves two major components: autoimmune-mediated inflammatory injury to the myelin sheath and neurodegeneration with progressive axonal loss. These two processes develop in parallel but with different dynamics throughout the disease and their interplay determines MS-related damage to CNS, with emerging clinical picture of this disorder^1^. Despite a substantial progress in understanding MS pathophysiology, some of its aspects have not been yet elucidated, including key elements of initiating immune-mediated inflammatory cascade in CNS or factors modulating highly variable course of the disease^2^. Thus, reliable markers are being extensively sought, which would allow to identify and predict activity and progression of the disease^3^. Investigations have long been focused mainly upon protein molecules as indices of immune activity or neuronal/axonal damage. However, with the advent of metabolomic approach, lipids have also gained attention in this field^4–7^. Sphingolipids (SL) seem to be promising candidates for the role of such biomarkers, as the main components of CNS myelin sheath^8^, but also relevant players in cellular signaling and cross-talk between inflammation and neurodegeneration^9^. Dysregulation of SL homeostasis exacerbates inflammatory responses, as well as promotes oxidative stress; these interrelated processes play an essential role in the etiology of CNS disorders, including MS^10^. There has been a solid evidence of accumulation of bioactive SL mediators, specifically ceramide (Cer), in different types of MS lesions and microstructurally altered CNS tissues. Cer and its glycosylated derivatives have been also revealed in the cerebrospinal fluid (CSF), serum, plasma and white blood cells of MS patients^7,11–14^.

Previous research by the members of our team indicated a significant role of Cer derivatives in initiation and shaping of the autoimmune response, targeted at CNS antigens, a key element of MS background. The analysis of the cellular and exosomal profile of bioactive SL mediators in human oligodendroglioma (HOG) cells showed that exosomal Cer subspecies (C16:0-, C24:0- and C24:1-Cer) participate in the apoptosis process induced by pro-inflammatory Th1 cytokines^15^. Comparative assessment of the lipid profile in brain tissues collected during autopsy (demyelinating plaques in the specimens from MS patients vs. unaffected cerebral white matter in the specimens from the control group) allowed to establish specific SL markers that differentiated active and inactive chronic MS lesions^16^. Active lesions were characterized by a significant increase in the main subclasses of dihydroceramide (dhCer), while inactive ones – by decreased levels of dhCer, Cer and sphingomyelin (SM) subclasses and significantly elevated levels of hexosylceramides (HexCer) and ceramide-1-phosphates (C1P). These findings suggested a key element of ‘pathological switch’ in the metabolic pathways of Cer, driving alternately active or inactive phase of the disease.

Furthermore, analysis of anti-Cer immunoglobulin G (IgG) in serum and CSF revealed elevated levels of their particular subclasses (anti-C16:0-, C18:0-, C18:1-, C24:0- and C24:1-Cer) in MS group in comparison with healthy subjects (HS) and those with other CNS disorders^17^. In addition, differences in anti-Cer IgG panels were observed between subgroups with different clinical types of MS [i.a. relapsing-remitting (RRMS) vs. progressive]. For selected anti-Cer IgG subclasses, significant correlations were found between their level in CSF and serum, as well as Link index. The results indicated the relevant role of Cer as a target for autoreactive humoral response, an essential element of MS pathology.

Encouraged by the above findings, which supported the need for further research aimed at identification of SL putative biomarkers for MS activity and progression, we undertook the current study focused on particular Cer derivatives. Our main goal was to evaluate the serum level of IgG antibodies against selected C1P subclasses in MS subjects in comparison with the reference groups. We also aimed at investigating their relationships with clinical status.

## Materials and methods

### Human specimens of serum

Participants were recruited among the patients hospitalized at the Department of Neurology, Wroclaw Medical University. The following 3 groups were included in this study: (i) patients with RRMS (*n* = 39), diagnosed according to McDonald’s criteria^18^; (ii) patients with other inflammatory disorders of the nervous system (I-OND, *n* = 13) that comprised chronic inflammatory demyelinative polyneuropathy (CIDP) or immune-mediated vasculitis, as well as (iii) patients with non-inflammatory disorders of the nervous system (NI-OND, *n* = 13), including migraine, cerebrovascular disease and hydrocephalus.

Exclusion criteria in RRMS and I-OND groups comprised: recent (within previous 3 months) short-term treatment with corticosteroids, any kind of long-term immunomodulatory or immunosuppressive treatment during at least previous 6 months, current infection and coexisting systemic diseases, in particular inflammatory ones. In the RRMS group, clinical data, such as duration of disease and degree of disability in Expanded Disability Status Scale (EDSS), have been determined based on medical records. The patients were divided into two subgroups, according to their clinical status: those in relapse – exacerbation of disease (RRMS-rel, *n* = 23) or in remission – a period of stable condition (RRMS-rem, *n* = 16).

In addition, the blood samples were obtained from healthy subjects (HS, *n* = 12) at the Regional Center of Blood Donation and Blood Treatment in Wroclaw.

Participation in the study was anonymous and voluntary. All the subjects gave informed written consent to participate in this study. The project was approved by the Bioethics Committee of Wroclaw Medical University (No. KB 337/2024). The study was conducted in accordance with the Declaration of Helsinki and Good Clinical Practice guidelines.

Collection of blood samples was conducted in the same clinical setting, in morning hours. In the patients with RRMS-rel or with exacerbation of I-OND, blood samples were collected prior to administration of corticosteroids or other immunosuppressive agents. Approximately 15 ml of peripheral venous blood was collected from each participant. After coagulation the samples were centrifuged at 2000xg at room temperature for 10 min, and afterwards stored at -80 °C prior to IgG preparation. All samples were coded and the subsequent analyses were carried out in a blind fashion in order to eliminate technical bias.

### Preparation of IgG antibodies from human sera

The IgG antibodies were isolated from human serum by affinity chromatography on Protein G GraviTrap columns (GE Healthcare). Prior to chromatographic separations, serum samples (0.5 ml) were centrifuged for 10 min at 10,000xg at 4 °C, then diluted 1:1 (v/v) with 20 mM phosphate buffer pH 7.0 and filtered through a 0.45 μm Millex-HV filter. Columns containing 1 ml of packed gel were equilibrated with phosphate buffer and prepared in the described above way sera were loaded. Proteins not bound by protein G were eluted from the columns with phosphate buffer. Next, the column was washed with 12 column volumes of phosphate buffer and the wash fraction was collected. Elution of IgG, bound to the ligand, was performed with 0.1 M Gly/HCl pH 2.7 and subsequently neutralized with 1 M Tris/HCl pH 9.0. During the fractionation the amount of proteins in all fractions was monitored by measuring the absorbance at 280 nm on 96 well UV half-plate area plates in an Enspire reader. The fractions containing IgG only were concentrated on an Amicon Ultra100K by ultracentrifugation at 4500xg at 4 °C to a final volume of 250 µl. During this process, IgG samples were desalted and albumin residues were removed. The protein concentration of column fractions was measured using bicinchoninic acid (BCA) protein assay kit (Pierce) with bovine serum albumin (BSA) as a standard. Purified IgG antibodies were preserved with 0.02% NaN_3_, aliquoted and stored at -80 °C prior to further analysis. They were analyzed individually.

### SDS-PAGE and Western blotting analysis

The electrophoretic separation was performed in a BIO-RAD Tetra Cell Systems apparatus as described earlier^17^. Briefly, proteins (2 µg) were analyzed on 12% polyacrylamide gel in reducing conditions and either stained with Coomassie Brilliant Blue (CBB) or transferred into 0.45 μm Immobilon P. The separated proteins were detected with, produced in donkey, anti-human IgG (H + L), conjugated with alkaline phosphatase (AP), antibody (1:7500) (Sigma). The reaction was visualized with AP substrates, according to standard procedure.

### Synthesis of C1P derivatives

C1P derivatives were synthesized in the Synthesis Unit of the Lipidomics Shared Resources at the Medical University of South Carolina, Charleston, SC, USA, as previously described^19^. Their purity was checked by the high performance liquid chromatography- tandem mass spectrometry (HPLC-MS/MS).

### ELISA assay

Before screening all purified IgG samples from patients examined as well as HS for their anti-C1P reactivity by ELISA assay, dilution linearity test was performed. The binding strength of purified human IgG antibody to 1 µg of C1P antigen was analyzed at ten different antibody concentration (1:1, 1:2.5, 1:5, 1:10, 1:25, 1:50, 1:100, 1:200, 1:300 and 1:400) as previously described^20^. Kinetic parameters, i.e., equilibrium constant Kd and Bmax, were determined by nonlinear regression of the obtained data, based on a monovalent antibody model using GraphPad Prism 7.01 software (GraphPad Software Inc., San Diego, CA, USA) (Supplemental Fig. S1, panel A). A very good dilution linearity, where response was proportional to the purified IgG concentration, was obtained in the range from 16.7 to 266.7 nM (Supplemental Fig. S1, panel B). The concentration of 166.5 nM was chosen for all IgG antibodies screening.

In the next stage of the study, an attempt was made to determine the antigen specificity of IgG antibodies from RRMS patients, individuals with OND and HS using an ELISA immunoassay. For this purpose, antibody binding to selected endogenous myelin antigens was examined: C16:0-C1P, C18:0-C1P, C18:1-C1P, C24:0-C1P, and C24:1-C1P. The methodological approach was as follows. Briefly, ELISA plates (Immulon 1B, Thermo Fisher Scientific) were coated with selected C1P solutions in absolute ethanol (10 µg/ml; 0.1 ml/well). The plates were incubated overnight at room temperature to facilitate complete solvent evaporation. Next, the plates were washed three times with phosphate buffered saline (PBS; 0.2 ml/well). Nonspecific binding sites were blocked by adding 100 µl of 1% (w/v) BSA in PBS and incubating the plates for 1 h at 37 °C. After washing 5 times with PBS purified IgG (0.1 mg/ml of protein, 0.1 ml/well) from serum of MS patients and individuals from reference groups were added and the plates were incubated overnight at 4 °C. Depletion controls (1% (w/v) BSA in PBS without IgG samples) were also included. In the next step the plates were washed again for 5 times with PBS and then incubated with horseradish peroxidase (HRP) conjugated anti-human IgG (GE Healthcare, 1:2000, 0.1 ml/well). The enzymatic activity was developed by adding peroxidase substrate (Sigma tablets). The colorimetric reaction was stopped by adding of 100 µl of 3 N H_2_SO_4_. and the optical density (OD) was read at 492 nm in an Enspire reader.

### Statistical analysis

Ordinary one-way Anova with post-hoc Tukey test was used to estimate differences in respect to age of examined subjects belonging to different groups.

Pairwise comparisons were conducted using the Mann–Whitney U test with Benjamini–Hochberg (BH) correction (α = 0.05). The BH correction is a standard procedure used to control the false discovery rate. Serum IgG anti‑C1P antibody levels were compared between patients with RRMS and the reference groups (HS, OND), as well as between the RRMS‑rel and RRMS‑rem subgroups. For each individual test, the adjusted p‑value and the effect size r were reported. The r measure was calculated as the ratio of the Z statistic (standardized test statistic) to the square root of N (the total sample size). The effect sizes were interpreted as small (0.1 ≤ *r* < 0.3), moderate (0.3 ≤ *r* < 0.5), and large (*r* ≥ 0.5). This statistical procedure was performed using the rstatix package in the R software^21^.

The diagnostic significance of immunoreactivities of these IgG antibodies with appropriate C1P antigens was analyzed using received operating characteristic (ROC) curves with clinical meaning considered as no discriminative (values from 0 to 0.5); limited (values from 0.5 to 0.7); moderate (values from 0.7 to 0.9) and high (> 0.9). The Youden index method was used for the determination of cut off points.

The IgG samples, for which the area under the curve (AUC) values were established as moderate or high, were subjected to a cluster analysis, a multivariate statistical method. Methods belonging to two different categories were used. A hierarchical Ward’s method was applied to determine the number of clusters. A non-hierarchical method, namely k-means clustering (k-means function of base R) from the selected anti-C1P IgG dataset was used for further analysis.

The Spearman’s rank correlation was used to check the associations between measured levels of anti-C1P antibodies and clinical parameters such as disease duration, EDSS and Link index.

The ROC analysis was performed using Statistica 13.3 software (StatSoft Inc., Tuls, OK, USA), cluster analysis was executed by OriginPro 2025b (OriginLab Corporation, Northampton, MA, USA). For the remaining analysis GraphPad Prism 7.01 was applied.

## Results

### Analysis of the study cohort

Basic demographic data, including age and sex structure of the patients included in the study are presented in the Table 1. One-way Anova followed by Tukey’s multiple comparisons test indicated no significant age differences between the study groups. In RRMS group, duration of the disease ranged from 1 to 108 months (mean 22 months) and EDSS score – from 1 to 4 (median 1.5) points. All the RRMS patients (*n* = 39) had magnetic resonance imaging (MRI) of the brain performed, with typical demyelinative lesions. In 32 patients the results of CSF testing were available, with confirmed presence of IgG oligoclonal bands and calculated Link index (increased value ≥ 0.7 in 20 cases).

**Table 1:** Basic demographics of the study subgroups.

Subgroup	Number of patients	Female/Male	Age (years) Mean ± SD (Min-Max)
RRMS-rel	23	16/7	32.2 **±** 10.1 (28–65)
RRMS-rem	16	6/10	33.1 **±** 7.5 (21–48)
I-OND	13	6/7	42.5 **±** 18.9 (22–83)
NI-OND	13	7/6	41.3 **±** 13.8 (19–64)

Abbreviations: I-OND-inflammatory other neurological diseases; NI-OND- non-inflammatory other neurological diseases; rel- relapse; rem- remission; RRMS- relapsing-remitting multiple sclerosis; SD- standard deviation.

### Analysis of Protein G-isolated and purified from human serum IgG antibodies

**Fig. 1:**
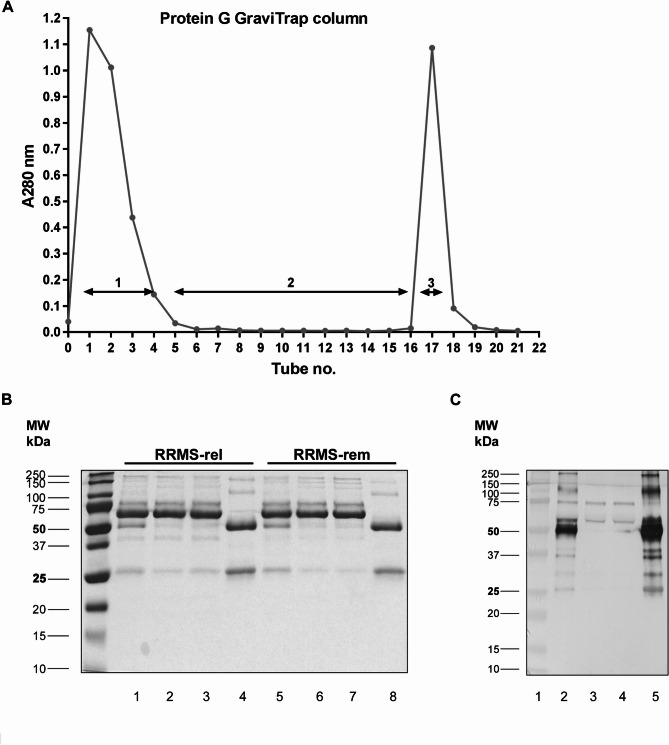
Purification of IgG antibodies from human serum. Fractionation of human serum on Protein G GraviTrap column **(panel A)**. Amount of 0.5 ml of serum, diluted 1:1 with 20 mM phosphate buffer, pH 7.0, was applied on the column (Fr. 0- load). This fraction was recycled 4 times (tubes [1-4]- Fr. 1- flow trough) and washed with phosphate buffer (tubes [5-16]- Fr. 2- wash). Bound IgGs were eluted with 0.1 M Gly/HCl buffer, neutralized with 1 M Tris/HCl, pH 9.0 (tube 17- Fr. 3- elution) and concentrated on an Amicon Ultra100K. Products present in the above fractions were analyzed on 12% SDS-PAGE and stained with 0.25% Coomassie Brillant Blue R-250 **(panel** **B)** or subjected to Western blotting analysis and immunostained with anti-human IgG (panel **C**). **Lane 1-** Fr.0, **lane 2-** Fr. 1, **lane 3-** Fr. 2, **lane 4-** Fr. 3. The molecular mass of the reference standards is shown on the left side. The Figure presents the cropped gel and blot; the original images are displayed in Supplemental Figure S2.

The IgG antibodies present in human serum were isolated by affinity chromatography on Protein G Gravi Trap columns. An example of preparation of IgG antibodies from 0.5 ml of serum is shown on Fig. 1. In a typical elution profile obtained during the isolation of IgG from RRMS-rel patient two peaks were visible (Fig. 1A). The first one − Fr. 1 − represented the proteins unbound by protein G. The second one, Fr. 3, corresponded to four isotypes of IgG (IgG_1_, IgG_2_, IgG_3_ and IgG_4_), bound by protein G. The obtained fractions were subjected to qualitative and quantitative analysis. The CBB staining (Fig. 1B) obtained as a result of electrophoretic separation of samples, collected during the isolation of serum IgG from both RRMS-rel and RRMS-rem, indicated that in both cases the products obtained as a Fr. 3 were IgG antibodies (lanes 4 and 8). This is evidenced by two characteristic bands with a molecular mass of 50 and 25 kDa, corresponding to the heavy and light chain of immunoglobulin G, as appeared under these conditions. In the remaining lanes (lanes 1–3 and 5–7), distinct bands with a molecular mass of about 65 kDa were observed, originating from other proteins found in the serum. Presumably, these bands indicate the presence of albumin, which constitutes up to 65% of all serum proteins, and perhaps the heavy chain of immunoglobulin M, constituting about 5 to 10% of serum immunoglobulins. Quantitation of proteins in the various fraction demonstrated high and comparable total protein amounts in Fr. 1 and residual protein amounts in Fr. 2. The preparation resulted in the recovery of 5.6 ± 0.9 mg of purified IgG (Fr. 3), a predominant class of antibody in serum, which corresponded to 14.3% ± 2.4 of all serum proteins (Supplemental Table S1). The absence of IgG in Fr. 1 and 2 (Fig. 1C; lanes 2 and 3, respectively) and the presence of the purified IgG antibodies was confirmed by Western blotting (Fig. 1C, lane 4). This analysis indicated the high efficiency of the IgG purification method used.

### Altered levels of serum anti-C1P IgG in RRMS patients

**Fig. 2:**
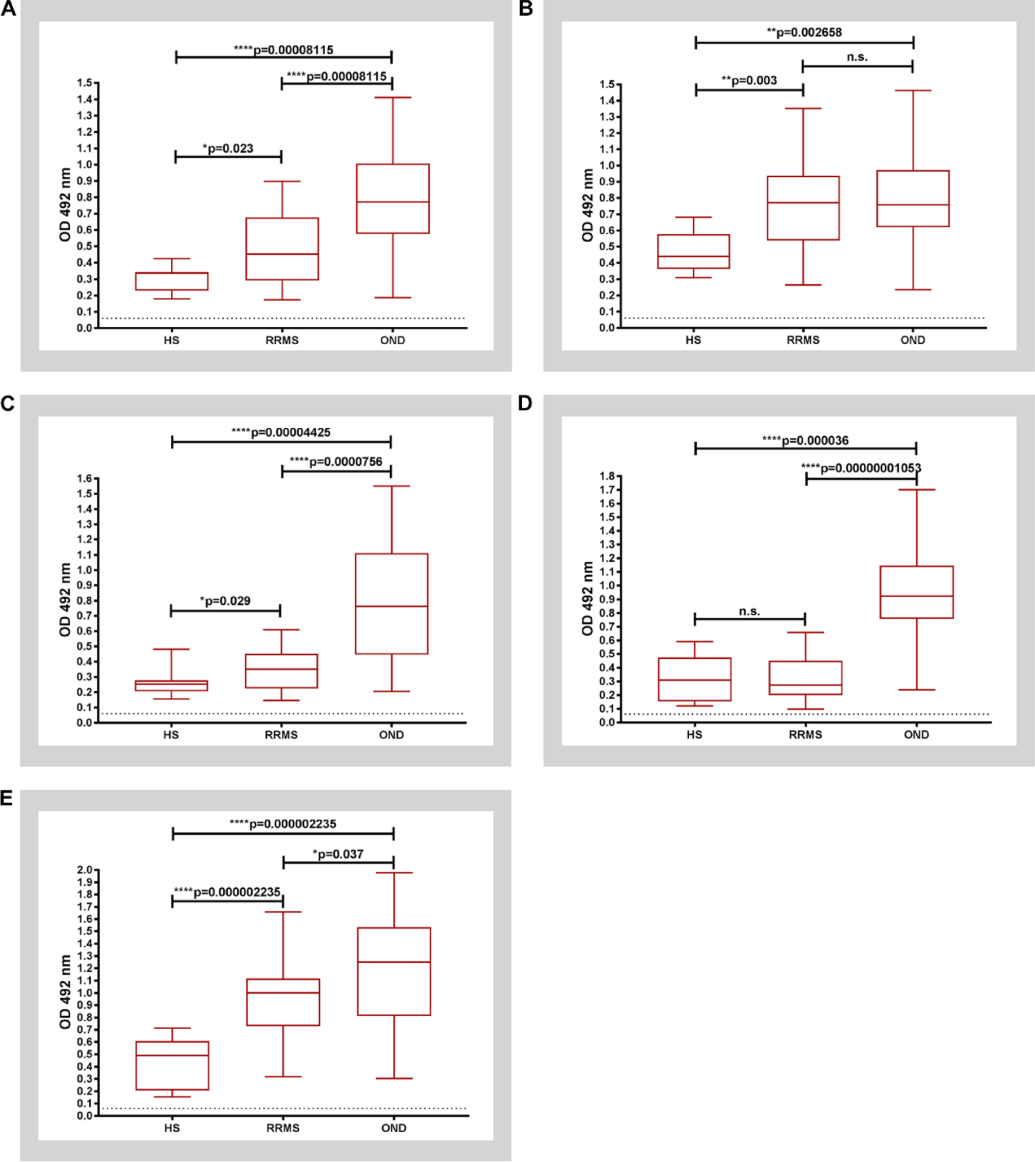
Reactivity of purified IgGs purified from human serum against: (**A**) C16:0-C1P, (**B**) C18:0- C1P, (**C)** C18:1- C1P, (**D**) C24:0- C1P and (**E**) C24:1- C1P by ELISA test. All boxes represent the 25th–75th percentile while horizontal lines inside of the boxes point out the median (50th percentile). Whiskers extend from the boxes indicate the range of the data - minimum and maximum values, accordingly. Depletion controls are indicated by dotted lines. Differences between groups were determined by the Mann–Whitney U test followed by Benjamini–Hochberg correction; adjusted p value is indicated. RRMS (*n* = 39), HS (*n* = 12), OND (*n* = 26). Abbreviations: HS- healthy subjects; n.s.- not significant; OND- other neurological diseases; RRMS- relapsing- remitting multiple sclerosis.

The Protein G-purified IgG antibodies from serum of MS patients (Fr. 3) were evaluated for the presence of selected C1P subclasses binding activity, using an ELISA (Fig. 2 and Supplemental Fig. S3).

The binding activity of serum IgG to C16:0-C1P was significantly higher in RRMS patients (median value: 0.4535) in comparison to HS group (median value: 0.3368) and significantly lower in relation to OND group (median value: 0.7725), with significance of adjusted p value (p_adj_) equals to p_adj_=0.023 and p_adj_=0.00008115, respectively (Fig. 2A).

The binding activity of serum IgG to C18:0-C1P in RRMS patients (median value: 0.7715) was significantly increased in comparison to HS group only (median value: 0.4395) with significance of p_adj_=0.003 (Fig. 2B). In turn, the binding activity of serum IgG to C18:1-C1P in RRMS individuals exhibited similar trend as to C16:0-C1P; appeared to be higher (median value: 0.35) than in HS group (median value: 0.252) with significance of p_adj_=0.029 and lower in relation to OND group (median value: 0.7628) with significance of p_adj_=0.00000756 (Fig. 2C).

RRMS patients manifested lower level of anti-C24:0-C1P serum IgG (median value: 0.2725) in comparison to OND group (median value: 0.924) with significance of p_adj_=0.00000001053 (Fig. 2D).

The binding activity of serum IgG to C24:1-C1P represented the pattern similar to C16:0-C1P and C18:1-C1P. Interestingly, serum IgG derived from RRMS patients exhibited the highest reactivity for C24:1-C1P among all C1P subclasses examined (median value: 1.001) in comparison to HS group (median value: 0.4915, p_adj_=0.000002235), while the level of anti-C24:1-C1P IgG was significantly decreased in RRMS in comparison to OND group (median value: 1.25; p_adj_=0.037) (Fig. 2E). The presented above results, obtained by ELISA test, along with their effect sizes and magnitudes, are summarized in Table 2.

**Table 2:** The levels of reactivity of selected anti-C1P serum IgG in relapsing-remitting multiple sclerosis (RRMS) patients in comparison to healthy subjects (HS) and individuals with other neurological diseases (OND).

Serum IgG	Group 1	Group 2	N1	N2	MEAN 1 ± SD	MEAN 2 ± SD	*P* value	Adjusted *p* value	Effect size	Magnitude
Anti-C16:0-C1P	RRMS	HS	39	12	0.4745 ± 0.21	0.3085 ± 0.08119	0.023	0.023	0.32	Moderate
	RRMS	OND	39	26	0.4745 ± 0.21	0.7838 ± 0.3031	0.0000541	0.00008115	0.50	Large
	OND	HS	26	12	0.7838 ± 0.3031	0.3085 ± 0.08119	0.0000476	0.00008115	0.66	Large
Anti-C18:0-C1P	RRMS	HS	39	12	0.7512 ± 0.2753	0.4714 ± 0.1202	0.002	0.003	0.43	Moderate
	RRMS	OND	39	26	0.7512 ± 0.2753	0.7789 ± 0.2951	0.725	0.725	0.04	Small
	OND	HS	26	12	0.7789 ± 0.2951	0.4714 ± 0.1202	0.000886	0.002658	0.52	Large
Anti-C18:1-C1P	RRMS	HS	39	12	0.3478 ± 0.1289	0.2555 ± 0.08237	0.029	0.029	0.31	Moderate
	RRMS	OND	39	26	0.3478 ± 0.1289	0.7749 ± 0.3728	0.00000252	0.00000756	0.58	Large
	OND	HS	26	12	0.7749 ± 0.3728	0.2555 ± 0.08237	0.0000295	0.00004425	0.68	Large
Anti-C24:0-C1P	RRMS	HS	39	12	0.3192 ± 0.1579	0.3219 ± 0.1633	0.903	0.903	0.02	Small
	RRMS	OND	39	26	0.3192 ± 0.1579	0.9394 ± 0.3448	0.00000000351	0.00000001053	0.73	Large
	OND	HS	26	12	0.9394 ± 0.3448	0.3219 ± 0.1633	0.000024	0.000036	0.69	Large
Anti-C24:1-C1P	RRMS	HS	39	12	0.9526 ± 0.3096	0.4265 ± 0.2061	0.00000134	0.000002235	0.61	Large
	RRMS	OND	39	26	0.9526 ± 0.3096	1.17 ± 0.4374	0.037	0.037	0.26	Small
	OND	HS	26	12	1.17 ± 0.4374	0.4265 ± 0.2061	0.00000149	0.000002235	0.70	Large

Pairwise comparisons were conducted using the Mann–Whitney U test with Benjamini–Hochberg correction. Abbreviations: C1P- ceramide-1-phosphate; SD- standard deviation.

Next, we reanalyzed the data considering the division of RRMS patients into subgroups in relapse and in remission (RRMS- rel and RRMS -rem) and OND patients – into subgroups with inflammatory or other etiology of disease (I-OND, NI-OND) (Supplemental Fig. S3). The Mann-Whitney U test with BH correction showed significantly higher levels of serum IgG derived from both RRMS subgroups only against C18:0-C1P compared to HS with significance p_adj_=0.018 in both cases (Supplemental Fig. S3 panel B, and Supplemental Table S3). In addition RRMS-rel exhibited higher level of serum anti-C24:1-C1P compared to HS (p_adj_=0.000333, Supplemental Fig. S3 panel E, and Supplemental Table S6).

The level of IgG anti-C16:0-C1P and anti-C18:1-C1P in RRMS-rel subgroup was significantly lower than in I-OND and NI-OND (Supplemental Fig. S3 panel A and Supplemental Table S2 as well as Supplemental Fig. 3S panel C, and Supplemental Table S4). In turn, RRMS-rem subgroup indicated decreased serum IgG anti-C16:0-C1P and anti-C18:1-C1P levels compared to NI-OND only (p_adj_=0.001155 and p_adj_=0.0000948, respectively) (Supplemental Fig. S3 panel A and Supplemental Table S2 as well as Supplemental Fig. S3 panel C, Supplemental Table S4, accordingly). The level of IgG anti-C24:1-C1P in in both phases RRMS-rel and RRMS-rem was significantly lower in relation to NI-OND only (p_adj_=0.00000408 and p_adj_=0.006, respectively) as it was illustrated in Supplemental Fig. S3 panel E and Supplemental Table S6. In turn, the level of IgG anti-C24:0-C1P in both RRMS subgroups was significantly lower than in I-OND and NI-OND (Supplemental Fig. S3 panel D, and Supplemental Table S5). The above results in details, including the effect sizes along with magnitudes, are included in Supplemental Tables S2-S6.

### ROC analysis

**Fig. 3:**
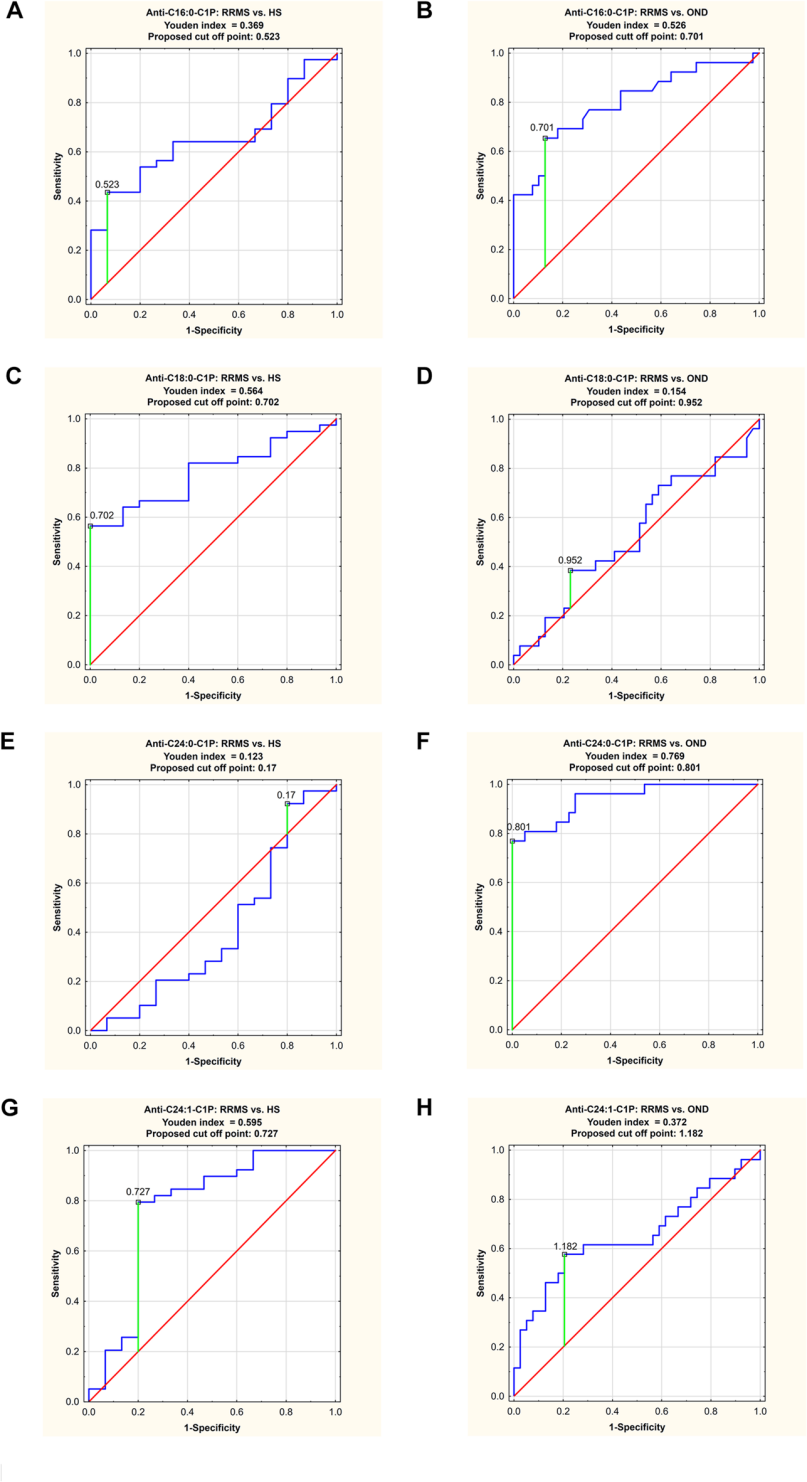
Receiver operating characteristic (ROC) curves for selected serum IgG anti-C1P antibodies with area under the curve (AUC) higher than 0.700. Abbreviations: C1P- ceramide-1-phosphate; HS- healthy subjects; RRMS- relapsing-remitting multiple sclerosis.

In order to validate ELISA results, we performed ROC analysis for all serum anti-C1P IgG levels in RRMS patients, as presented in Table 2. The results of ROC curve analysis for anti-C1P IgG data sets with the AUC value higher than 0.700 are presented in Fig. 3. The cut-off points, determined by the Youden index method, are indicated in Table 3.

**Table 3:** Summary of receiver operating characteristic (ROC) curves for serum anti-C1P IgG levels in the study groups.

Antibody	Examined groups	AUC	AUC with 95% confidence interval	Youden index	Cut off point	Sensitivity	Specificity	ACC	PPV	NPV	*P* value
Anti-C16:0-C1P	RRMS vs. HS	0.653	0.506–0.800	0.369	0.523	0.436	0.933	0.574	0.944	0.389	0.0417
	**RRMS vs. OND**	**0.798**	**0.682–0.914**	**0.526**	**0.701**	**0.654**	**0.872**	0.785	0.773	0.791	**0.0000**
Anti-C18:0-C1P	**RRMS vs. HS**	**0.781**	**0.661–0.904**	**0.564**	**0.702**	**0.564**	**1.000**	0.685	1.000	0.469	**0.0000**
	RRMS vs. OND	0.532	0.385–0.679	0.154	0.952	0.385	0.769	0.615	0.526	0.652	0.6682
Anti-C18:1-C1P	RRMS vs. HS	0.569	0.381–0.758	0.400	0.297	0.667	0.733	0.685	0.867	0.458	0.4718
	RRMS vs. OND	0.153	0.047–0.258	0.654	0.619	0.000	0.346	0.138	0.000	0.188	0.0000
Anti-C24:0-C1P	RRMS vs. HS	0.409	0.222–0.595	0.123	0.17	0.923	0.200	0.800	0.722	0.750	0.3371
	**RRMS vs. OND**	**0.942**	**0.886–0.998**	**0.769**	**0.801**	**0.769**	**1.000**	0.908	1.000	0.867	**0.0000**
Anti-C24:1-C1P	**RRMS vs. HS**	**0.769**	**0.603–0.936**	**0.595**	**0.727**	**0.795**	**0.800**	0.796	0.912	0.600	**0.0015**
	RRMS vs. OND	0.654	0.508–0.800	0.372	1.182	0.577	0.795	0.708	0.652	0.738	0.0386

Abbreviations: ACC-accuracy; AUC- area under the curve; C1P- ceramide-1-phosphate; HS- healthy subjects; NPV-negative predictive value; OND- other neurological diseases; PPV-positive predictive value; RRMS- relapsing-remitting multiple sclerosis.

Anti-C16:0-C1P IgG level indicated a moderate value in differentiating RRMS group from OND group (Fig. 3B), with sensitivity 65.4% and specificity of 97.2%, respectively (proposed cut off point: 0.701, AUC = 0.798). Anti-C18:0-C1P IgG level showed moderate discriminating value between RRMS and HS groups (Fig. 3C), with sensitivity and specificity of 56.4% and 100%, respectively (proposed cut off point: 0.702, AUC = 0.781). The level of anti-C24:0-C1P IgG had a high discriminating value between RRMS group and OND group, with sensitivity of 76.9% and specificity of 100%, respectively (proposed cut off point: 0.801, AUC = 0.942) (Fig. 3F). The level of anti-C24:1-C1P IgG exhibited a moderate value in differentiating between RRMS group and HS group, with sensitivity of 79.5% and specificity of 80% (proposed cut off point: 0.727, AUC = 0.769) (Fig. 3G). These results are shown in Table 3.

### Cluster analysis

**Fig. 4:**
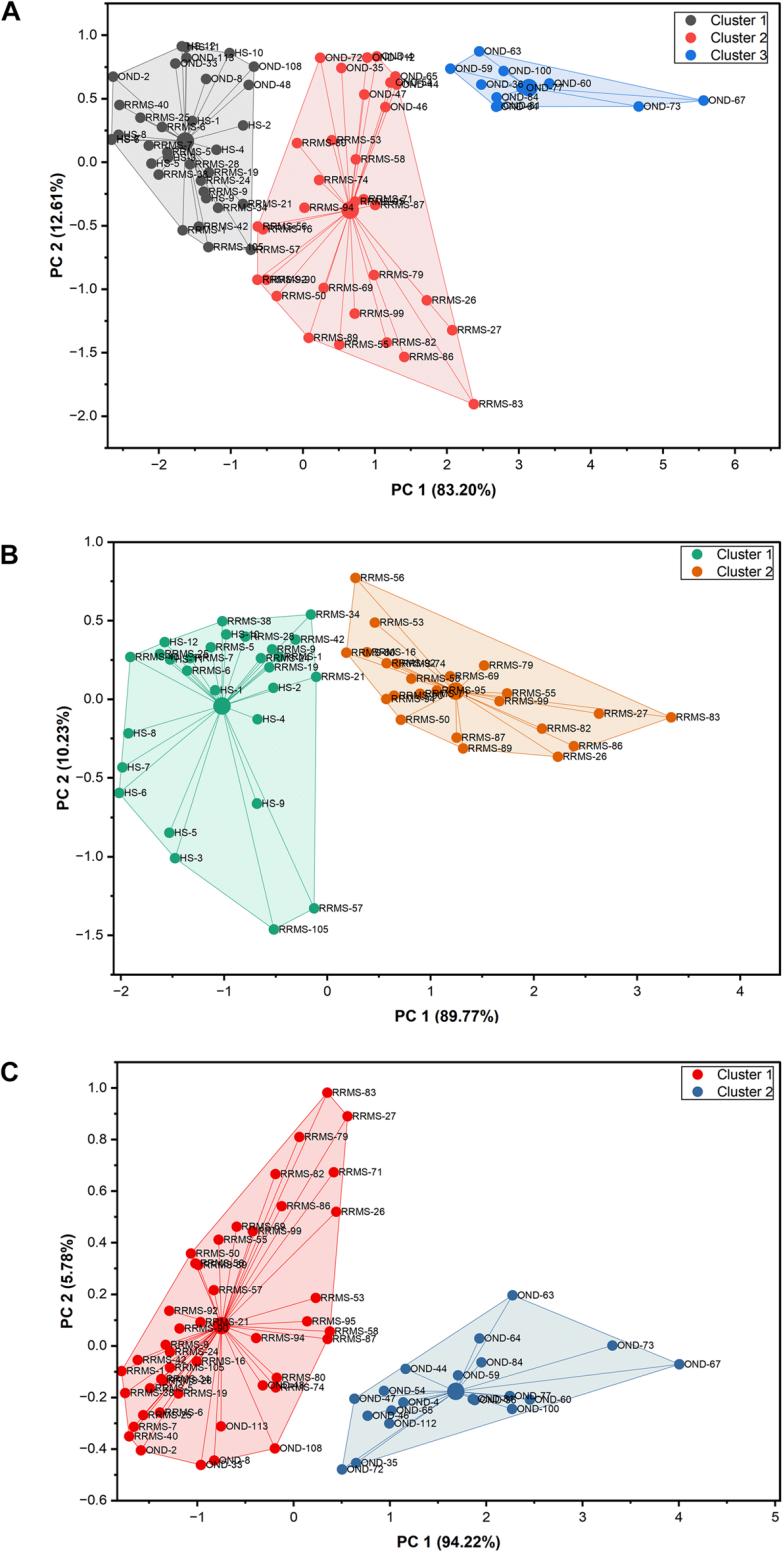
Visualization of the cluster plots with Convex Hull in a two dimensional space defined by the two most significant Principal Components. The assignments were computed from the original dataset differentiating: (**A)** RRMS, HS and OND group and containing observations related with anti-C16:0-C1P, anti-C18:0-C1P, anti-C24:0-C1P, and anti-C24:1-C1P IgG levels; (**B)** RRMS and HS group and containing observations related with anti-C18:0-C1P and anti-C24:1-C1P IgG levels; and (**C)** RRMS and OND group and containing observations related with anti-C16:0-C1P and anti-C24:0-C1P IgG levels, respectively using k-means algorithm. Next, the data were reduced into a two-dimensional space using the Principal Component Analysis (PCA). Different colors of dots represent assignment of a given observation to the respective cluster. Abbreviations: HS- healthy subjects; OND- other neurological diseases; RRMS- relapsing- remitting multiple sclerosis.

Based on the results of ROC analysis, serum anti-C1P IgG levels with AUC equal or greater than 0.769 were selected to perform cluster analysis. The number of expected clusters was pre-determined by using the hierarchical Ward’s method (Supplemental Fig. S4). Relative level of anti-C16:0-, anti-C18:0-, anti-C24:0- and anti-C24:1-C1P serum IgG was subsequently used to perform k-means clustering to categorize the samples examined into 3 clusters (Fig. 4A). Cluster 1 comprised 34 subjects: all healthy ones; 16 patients with RRMS (including 9 ones from RRMS-rel subgroup) and 6 individuals with OND (containing 5 from I-OND subgroup). Cluster 2 included 23 RRMS patients (14 from RRMS-rel, 9 from RRMS-rem subgroup) and 9 OND ones (5 from I-OND and 4 from NI-OND subgroup). Cluster 3 consisted of OND patients only, with the majority belonging to NI-OND subgroup (8 out of 11) (Supplemental Fig. S5).

Significant differences in anti-C1P IgG panels between the study groups (RRMS vs. HS: anti-C18:0- and anti-C24:1-C1P IgG; RRMS vs. OND: anti-C16:0- and anti-C24:0-C1P IgG) (Fig. 2), with discriminatory ability of these IgG subsets, confirmed by ROC analysis (Fig. 3; Table 3), together with similar patterns of relative levels of anti-C16:0- / anti-C24:0-C1P, as well as anti-C18:0- /anti-C24:1-C1P IgG (Supplemental Fig. S6), supported the notion that anti-C1P IgG panels might differentiate pathological from physiological condition but also reflect distinct biological processes (i.e. neuroinflammation vs. neurodegeneration) with different degrees of sensitivity. To check this hypothesis, a k-means analysis was subsequently performed to divide the appropriate IgG samples into 2 clusters. Relative levels of anti-C18:0- and anti-C24:1C1P serum IgG were subjected to k-means clustering to differentiate RRMS from HS group (Fig. 4B). Cluster 1 comprised 28 subjects (12 HS and 16 RRMS, almost equally distributed between RRMS-rel and RRMS-rem subgroups) whereas cluster 2 was represented by 23 RRMS patients (mainly RRMS-rel subgroup) (Supplemental Fig. S7). In turn, relative levels of anti-C16:0- and anti-C24:0-C1P serum IgG were subjected to k-means clustering to differentiate RRMS from OND group (Fig. 4C). Cluster 1 comprised 45 subjects: all RRMS patients (*n* = 39) and 6 OND ones (mainly from I-OND subgroup), whereas cluster 2 was composed of 20 OND subjects, mainly from NI-OND subgroup (Supplemental Fig. S8).

### Correlation analysis between selected serum anti-C1P IgG derived from RRMS patients and clinical parameters

Next, we examined whether there are any correlations between anti-C1P IgG titers (as reflected by OD at 492 nm) in RRMS group and MS-related clinical parameters. The levels of anti-C16:0-C1P, anti-C18:0-C1P, anti-C18:1-C1P, anti-C24:0-C1P and anti-C24:1-C1P IgG in RRMS did not correlate with disease duration (Supplemental Fig. S9, panels A, D, G, J and M) or EDSS score (panels B, E, H, K and N), accordingly. No associations were found between the levels of these antibodies and Link index, either (Supplementary Fig. S9, panels C, F, I, L and O, respectively). The summary of this analysis is displayed in Table 4.

**Table 4:** Correlation of serum anti-C16:0-C1P, anti-C18:0-C1P, anti-C18:1-C1P, anti-C24:0-C1P, anti-C24:1-C1P IgG levels in RRMS group with clinical parameters.

Serum IgG	Clinical parameter	Spearman’s correlation coefficient predictor (*r* value)	*P* value
Anti-C16:0-C1P	Disease duration	-0.1753	0.2857
	EDSS	0.04531	0.7842
	Link index	-0.1286	0.4759
Anti-C18:0-C1P	Disease duration	-0.2746	0.0906
	EDSS	0.06427	0.6975
	Link index	0.0565	0.7548
Anti-C18:1-C1P	Disease duration	-0.251	0.1232
	EDSS	0.08361	0.6128
	Link index	-0.2063	0.2494
Anti-C24:0-C1P	Disease duration	-0.1884	0.2506
	EDSS	-0.01253	0.9396
	Link index	-0.2496	0.1613
Anti-C24:1-C1P	Disease duration	-0.1706	0.2992
	EDSS	0.03706	0.8228
	Link index	-0.1914	0.2859

Abbreviations: C1P- ceramide-1-phosphate; EDSS- Expanded Disability Status Scale; RRMS- relapsing-remitting multiple sclerosis.

## Discussion

Cer products have been suggested to be involved in the main mechanisms underlying MS pathology, including those - recently highlighted - driven by B cells. Activation of B cells together with cytokine stimulation promote tissue penetration and destabilization of membrane lipids in CNS, resulting in damage to myelin sheath. SL (in particular Cer derivatives) are essential for maintenance of lipid membranes integrity, so changes in their metabolism and proportion correspond with dynamic processes of demyelination and repair and their pathophysiological consequences^22^. Although in the aforementioned studies there is convincing evidence for Cer abnormalities in MS, it relies mainly on preclinical models and their translation into human clinical context, with clear identification of Cer related mechanisms of action and potentially relevant MS biomarkers, still remains a challenge^4–7,23^. Based on results of our previous studies^15–17^ and scarce literature data^11,13,14,24,25^, here we focused on particular Cer derivative – C1P, aiming to investigate its putative role as a target and/or mediator of autoimmune response in MS.

C1P, a major metabolite of Cer, can be formed through a stereospecific reaction of phosphorylation, due to direct action of Cer kinase (CerK), while the reverse reaction (conversion of C1P to Cer) is mediated by C1P phosphatase. An appropriate balance between these two molecules is required to ensure cells and tissues homeostasis through regulation of relevant metabolic or signaling pathways. Thus, coordinated action of CerK and C1P phosphatases may have crucial importance for maintaining this balance. Its disturbances with a shift towards accumulation either Cer or C1P may result in metabolic dysregulation, supposedly involved in the background of autoimmune diseases^26^, neurodegenerative disorders^27^, cardiovascular illnesses^28^ or cancer^29^.

It has been demonstrated that C1P exerts pro-inflammatory properties; however, the underlying mechanisms have not been yet fully elucidated. One of the major function of C1P is its capability to activate cytosolic phospholipase PLA_2_ (cPLA_2_), which mediates release of arachidonic acid^30^- the substrate used for production of eicosanoids, such as prostaglandin E_2_ (PGE_2_). This is aligned with the fact that MS patients exhibit higher PGE_2_ expression/activity in CSF^31^, lymphocytes^32^ as well as demyelinative lesions in brain tissue^33^. A sharp increase of this pro-inflammatory mediator was observed in active stage of MS^34^, and experimental studies with the use of cyclooxygenase (COX) inhibitors, which oppose C1P action by preventing PGE_2_ synthesis, suggested their beneficial effect as potential options in MS therapy^35^.

Other pro-inflammatory activities affected by C1P include: activation of degranulation in mast cells^36^, stimulation of phagocytosis in neutrophils^37^, as well as stimulation of macrophage migration^38^. C1P also increases transport of P-glycoprotein, which regulates the permeability of the blood brain barrier via COX-2/PGE_2_ signaling^39^.

On the other hand, it has been shown that C1P might also act as an anti-inflammatory agent, under specific conditions. Anti-apoptotic effects of C1P, promoting cell survival, are related with its ability to block activity of serine palmitoyltransferase^40^, acid sphingomyelinase^41^ and TNF-α^42^. Furthermore, it promotes macrophage chemoattractant protein-1 (MCP-1) release in different types of cells^38^. Taken together, C1P seems to act on its own rights, executing regulatory effects and modulating cell functions depending on circumstances.

Although C1P has been reported to exert anti-inflammatory and homeostatic functions in neural tissues, accumulating evidence indicates that SL metabolism is profoundly altered during neuroinflammatory and neurodegenerative processes. Dysregulation of Cer–SL pathways has been widely documented in MS and other neurodegenerative disorders, including alterations in Cer, sphingosine-1-phosphate, and related lipid mediators, which are tightly linked to immune activation and neuroinflammation^10,43–47^. C1P itself is recognized as a bioactive lipid involved in the regulation of inflammatory signaling pathways, immune cell migration, and phospholipase A2 activation, placing it at the interface between lipid metabolism and immune regulation^38,48,49^. We therefore propose that increased anti-C1P IgG levels observed in RRMS patients reflect a secondary immune response to neuroinflammation-driven alterations in SL metabolism rather than a primary pathogenic mechanism. Local dysregulation of Cer and C1P turnover in the inflamed CNS may lead to abnormal exposure or release of C1P-containing membrane domains, promoting the formation of lipid neoepitopes and subsequent induction of a humoral immune response. This interpretation is further supported by previous reports demonstrating the presence of autoantibodies against lipid and glycolipid antigens in MS and other neuroinflammatory diseases, including antibodies directed against phospholipids, gangliosides, sulfatides, and myelin-associated lipid complexes^50–52^. In this context, anti-C1P antibodies are more likely to represent biomarkers of active neuroinflammatory processes rather than direct drivers of disease pathology.

In one of the previous reports by our team members, an increase in C1P subspecies (mainly C18:1-C1P and C24:1-C1P) was demonstrated in inactive MS lesions in comparison with specimens representing other CNS disorders or normal brain tissue. On the contrary, active MS plaques were characterized by a significant decrease of C1P subspecies^16^. The studied autopsy samples from MS subjects represented advanced stage of disease, associated with chronic inflammation and presumably extensive neurodegenerative component. In the present study we focused on earlier phase of the disease (i.e. RRMS), when active immune-mediated inflammation is a predominating mechanism of CNS injury - therefore antibodies as markers of autoimmune humoral response were chosen as the subject of investigation. The main findings presented here included elevated levels of nearly all analyzed anti-C1P IgG in serum of RRMS patients in comparison to HS, with the highest computed effect size (*r* = 0.61), denoted in Table 2 as large, for anti-C24:1-C1P (Fig. 2E). There is a striking consistency in the results from both studies, despite differences in MS stages and studied material (brain tissues vs. serum). Translation of metabolomic profiles in autopsy tissue samples into presence of targeted antibodies in body fluids from patients with active disease is extremely complex and requires analysis of many associated factors (i.a. changing dynamics of metabolic processes in the course of disease and compartmentalization of inflammatory process within CNS during its late stages). It can be hypothesized that each stage of MS is associated with the concerted action of Cer derivatives (perhaps with most relevant role of C1P), with their changing interactions in this pathological cross-talk.

It should be mentioned that the level of IgG anti-C24:1-C1P was significantly lower in both RRMS subgroups in relation to NI-OND only (Supplemental Fig. S3, panel E), whereas the level of IgG anti-C24:0-C1P in both RRMS subgroups was significantly lower than in both OND subgroups (Supplemental Fig. S3, panel D). Overall, these results suggest that increased humoral response against long-chain C1P subspecies is particularly specific for MS and it does not just reflect overall neuronal injury or dysregulated immune-mediated inflammation in CNS. However, comparisons between RRMS and OND subgroups have to be interpreted cautiously. I-OND subgroup comprised patients with immune-mediated disorders (vasculitis and chronic polyneuropathy), which share inflammatory background with MS but differ in source of autoreactive humoral response (intrathecal Ig synthesis in MS). NI-OND subgroup was more heterogenous (migraine, cerebrovascular disease and hydrocephalus) which might have affected the results in view of small sample size. In further exploration, involvement of larger groups representing particular neurological conditions, with or without inflammatory component, would enable more precise analysis of these relationships. Importantly, the reliability of the above findings was supported by eliminated impact of immunosuppressive/immunomodulatory drugs, as the patients were included in the study prior to such treatment or after appropriate wash-out period.

There is an evidence that fatty acid chain length of Cer can determine biophysical properties of this SL mediator^53^. In physiological conditions, medium chain Cer (C16 and C18) are known to be enriched in cerebral gray matter, while long chain ones (C24) - in white matter and myelin sheath^54^. Considering distribution of Cer derivatives in the brain, presence of the medium chain ones would reflect neuronal injury^55^, while the long chain ones – oligodendrocytes degradation^15^. The long chain Cer (C24) are also known to be the major Cer subspecies present in serum/plasma whereas C1P constitute only 0.15% of all SL components^56^. However, it should be taken into account that the pattern of anti-Cer IgG in body fluids, revealed by our previous study^17^ reflected metabolic perturbations of Cer, supposed to occur due to processes involved in MS background. The anti-Cer IgG alterations in serum (unlike in the CSF) were observed only for Cer containing medium chains (C16:0-, C18:0- and C18:1-Cer) with no change for the long chain ones^17^. Current findings revealed altered patterns of IgG against C1P in serum of RRMS patients. Here we found that anti-C16:0-C1P (Fig. 2A), anti-C18:0-C1P (Fig. 2B), anti-C18:1-C1P (Fig. 2C) and anti-C24:1-C1P (Fig. 2E) serum IgG levels were increased in RRMS patients in comparison with HS group. In turn, levels of IgG against C16:0-C1P (Fig. 2A), anti-C18:1-C1P (Fig. 2C), C24:0-C1P (Fig. 2D) and C24:1-C1P (Fig. 2E) were decreased in RRMS in relation to OND (including distinguished subgroup of inflammatory ones). There were not significant differences between RRMS and HS group in the case of anti-C24:0-C1P (p_adj_=0.903) and between RRMS and OND for anti-C18:0-C1P (p_adj_=0.725). What is more, the calculated effect sizes were negligible (*r* < 0.04) which would suggest some similarities between groups. Nevertheless, due to small sample sizes considered in this study, this phenomenon requires further investigations. In addition, the results of ROC analysis indicated that the determination of anti-C18:0-C1P and anti-C24:1-C1P serum IgG levels might be useful for discriminating RRMS from HS group (Fig. 3, panel C and G), whereas anti-C16:0-C1P and anti-C24:0-C1P serum IgG levels – for discriminating RRMS from OND group (Fig. 3, panel B and F). In these cases, there were relatively high values of PPV− between 0.773 and 1.0 (see Table 3). They suggest that proposed classifiers have a strong tendency to decrease the level of false positive errors meaning that less subjects will be inappropriately diagnosed with RRMS. Furthermore, the usefulness of the above anti-C1P IgG subsets in distinguishing pathological from physiological conditions, as well as disorders with different etiology, was confirmed by means of multivariate statistical method of cluster analysis (Supplemental Fig. S7 and S8). Despite somehow subjective selection of variables for clustering and the number of clusters (justified by the results of initial analysis), the findings from this multivariate technique support the concept that panels/sets of anti-C1P IgG may be of greater discriminating value than single immunological parameters (Fig. 4). Such a panel including anti-C16:0-C1P, anti-C18:0-C1P, anti-C24:0-C1P and anti-C24:1-C1P IgG might be considered for further investigation towards potential clinical implications - the use of anti-C1P IgG as biomarkers in differential diagnosis at MS onset, especially in atypical cases.

In recent years, specific autoantibodies (IgG anti-AQP4 and anti-MOG) have been revealed as pathological hallmarks specific for neuromyelitis optica spectrum disorders (NMOSD) and anti-MOG Abs associated disease (MOGAD), currently used as their core diagnostic biomarkers. However, no such biomarkers have been identified for MS so far. Neurofilaments light chains (Nfl) and glial fibrillary acidic protein (GFAP) are considered as reliable indicators of MS activity and/or progression but their specificity is very low as they reflect neuronal injury and astroglial reactivity in various CNS diseases, independent from their background. Thus additional biomarkers helpful in discriminating MS from other CNS diseases are being extensively sought^8^.

Few attempts have been made to use anti-Cer antibodies as biomarkers of activity in other disorders, including those of the nervous system. In leprosy, presence of anti-Cer antibodies was found to be related to extent of nerve sheath damage and particular bacterial strains as pathogenic factors^57^. In the rare autoimmune condition, encephalomyeloradiculoneuropathy (EMRN), accumulation of species-specific Cer (mainly long chain ones) was demonstrated in CSF and presence of anti-neutral glycolipids antibodies – in CSF and serum. Profile of these antibodies allowed to differentiate patients with ERMN from the reference group with Parkinson disease, and their titers decreased after effective immunomodulatory treatment. Dysregulation of glycolipids metabolism and its impact upon invariant natural killer T cell development, as well as abnormal complement activation, were suggested to play a relevant role in EMRN pathophysiology^54,58^. Furthermore, in non-small-cell lung cancer (NSCLC) anti-Cer antibodies were supposed to be produced by the tumor and its microenvironment, and to neutralize pro-apoptotic effect of Cer. Significantly elevated levels of anti-Cer antibodies were revealed in serum and bronchial wash fluid samples from NSCLC group (in comparison with HS) and they showed a tendency (though not significant) for prolonged overall survival^59^.

Evaluation of activity in MS has been so far based on clinical assessment and radiological indices (new and/or contrast-enhanced lesions in MRI). However, there is an ongoing search for specific and reliable body fluid biomarkers of disease activity^8^. In view of our previous findings, Cer derivatives and antibodies against them seemed promising candidates for the role of such biomarkers. Following more precise analysis of RRMS subgroups, no significant differences in anti-C1P IgG levels were found between those in relapse or in remission. No correlations were revealed between these IgG levels and disease duration, EDSS score and Link index, either.

Although no relationships were found in our study group between immunological measures and MS-related clinical data (Supplemental Fig. S9), potential clinical utility of these findings should not be discredited. The group was characterized by a relatively short MS duration and mild disability, and marked individual differences in disease course in MS populations must be taken into account. Perhaps in a larger group of patients and in prospective observation these relationships would appear more relevant.

Indeed, a small sample size constitutes a limitation of our study, with regard to the study design including comparative analysis of multiple subgroups. However, statistical models applied in the analyses were meant to mitigate these constraints. Furthermore, because of focusing on RRMS only, the results of the study may not accurately reflect abnormalities in SL metabolic pathways in different types and stages of the disease. Another limitation is associated with the fact that anti-C1P IgG presence was determined only in serum and not in CSF. Nevertheless, in view of long-term course of MS and need for regular follow-up, serum/plasma biomarkers are much desired due to their better availability. Based on experiences with other body fluid biomarkers, their serum level may adequately reflect their production in CNS^8^. There is also some evidence that peripheral blood may contribute to formation of immune complexes due to selective transport and the blood-brain barrier (BBB) leakage^60^.

To our best knowledge, this study for the first time focuses on the presence and meaning of anti-C1P antibodies in serum of MS patients. Strengths of the study include also complex statistical approach (combined ROC and cluster analysis) which allowed comprehensive presentation of relevant findings, as well as evaluation of links between immunological and clinical data. These results shed some light on the perturbation on SL metabolism (especially significance of balance between Cer and C1P) in the background of neurological diseases, specifically MS, encouraging further targeted investigations in this field. Our findings support the concept of altered Cer metabolism as a target and/or mediator of autoimmune response in MS. Nevertheless, potential value of antibodies anti-C1P subclasses as diagnostic biomarkers or measures of MS activity warrants verification in larger groups of MS patients, preferably in prospective observation. Furthermore, profile of selected anti-C1P IgG should be assessed parallel in blood and CSF, with thorough analysis of links between these two compartments and their contributing factors. Hopefully, these studies will lead to a better understanding of the role of this intriguing phosphosphingolipid in pathophysiology of MS, with emerging clinical implications.

## Conclusions

The serum levels of IgG against C18:0-C1P and C24:1-C1P subspecies differed significantly between the patients with RRMS and HS, whereas anti-C16:0-C1P and anti-C24:0-C1P IgG - between the patients with RRMS and those with OND, with large effect size in all above cases, validated also by ROC analysis. These findings suggest the relevant role of neuroinflammation-driven alterations in Cer metabolism (particularly C1P) as a target and/or mediator of autoimmune humoral response in MS. Potential value of panels of anti-C1P antibodies as biomarkers in differential diagnosis of MS warrants further investigations (including multi-center studies with larger MS cohorts and their prospective observation).

## Supplementary Information

Below is the link to the electronic supplementary material.

Supplementary Material 1

## Data Availability

The datasets used and/or analyzed during the current study are available from the corresponding author on reasonable request.

## Abbreviations

ACC: Accuracy
AP: Alkaline Phosphate
AUC: Area Under Curve
BBB: Blood- Brain Barrier
BCA: Bicinchoninic Acid
BSA: Bovine Serum Albumin
C1P: Ceramide-1-Phosphate
CBB: Coomassie Brilliant Blue
Cer: Ceramide
CerK: Ceramide Kinase
CIDP: Chronic Inflammatory Demyelinative Polyneuropathy
CNS: Central Nervous System
COX-2: Cyclooxygenase-2
cPLA_2_: Cytosolic Phospholipase A_2_
CSF: Cerebrospinal Fluid
EAE: Experimental Autoimmune Encephalomyelitis
EDSS: Expanded Disability Status Scale
ELISA: Enzyme-Linked Immunosorbent Assay
EMRN: Encephalomyeloradiculoneuropathy
HexCer: Hexosylceramide
HOG: Human Oligodendroglioma
HPLC-MS/MS: High Performance Liquid Chromatography- tandem mass spectrometry
HRP: Horseradish Peroxidase
HS: Healthy Subjects
IgG: Immunoglobulin G
I-OND: Inflammatory Other Neurological Diseases
MCP-1: Macrophage Chemoattractant Protein-1
MRI: Magnetic Resonance Imaging
MS: Multiple Sclerosis
NI-OND: Non- Inflammatory Other Neurological Diseases
NPV: Negative Predictive Value
N.S: Not significant
NSCLC: Non-Small-Cell Lung Cancer
OD: Optical Density
OND: Other Neurological Diseases
P_adj_: Adjusted p value
PBS: Phosphate Buffered Saline
PC: Principal Component
PGE_2_: Prostaglandin E_2_
PPV: Positive Predictive Value
Rel: Relapse
Rem: Remission
ROC: Received Operating Characteristic
RRMS: Relapsing- Remitting Multiple Sclerosis
SD: Standard Deviation
SDS-PAGE: Sodium Dodecyl Sulfate-Polyacrylamide Gel Electrophoresis
SL: Sphingolipid
SM: Sphingomyelin
