# Supplementary material for: A potential of serum anti-C1P IgG antibodies as biomarkers in differential diagnosis of relapsing-remitting multiple sclerosis
Source: Sci Rep. 2026 Mar 19;16:9437. doi: 10.1038/s41598-026-43823-y (PMC13002994; doi:10.1038/s41598-026-43823-y)
Supplement: Supplementary file 1 — Supplementary Material 1 [file 41598_2026_43823_MOESM1_ESM.pdf]

Supplementary Information

A potential of serum anti-C1P IgG antibodies as biomarkers in differential diagnosis of relapsing-remitting multiple sclerosis

Justyna Chojdak-Lukasiewicz[1,#], Anna Jakubiak-Augustyn[2,#], Zdzislaw M. Szulc[4], Jerzy Gubernator[2], Pawel Blazej[3], Anna Pokryszko-Dragan[1], Slawomir Budrewicz[1], Maria Podbielska[4,5*]

From: [1]Department of Neurosurgery and Neurology, Wroclaw Medical University, Borowska 213, 50-556 Wroclaw, Poland;

[2]Department of Lipids and Liposomes and [3]Department of Bioinformatics and Genomics, University of Wroclaw, F. Joliot-Curie 14a,50-383 Wroclaw, Poland;

[4]Department of Biochemistry & Molecular Biology, Medical University of South Carolina, 173 Ashley Avenue, Charleston, SC 29425-2503, USA;

[5]Laboratory of Microbiome Immunobiology, Ludwik Hirszfeld Institute of Immunology & Experimental Therapy, Polish Academy of Sciences, Rudolfa Weigla 12, 53-114 Wroclaw, Poland

Running title: Anti-C1P antibodies in RRMS

[#]These authors contributed equally to this study

*To whom correspondence should be addressed: Maria Podbielska, Ludwik Hirszfeld Institute of Immunology & Experimental Therapy, Polish Academy of Sciences, Laboratory of Microbiome Immunobiology, Rudolfa Weigla 12, 53-114, Wroclaw, Poland; Tel: +48-71-370-99-12, Fax: +48-71-337-21-71, E-mail: maria.podbielska@hirszfeld.pl

Keywords: biomarkers, ceramide, C1P, lipid antigens, multiple sclerosis, sphingolipids.

Fraction no.	Specification	Preparation stage	Fraction volume (ml)	Proteins present	Protein concentration ± SEM (mg/ml)	Mean yield ± SEM * (%)
Fr. 0	Load	Before affinity	1	Albumin, globulins	38.8 ± 0.8	–
Fr. 1	Flow-through	After affinity	4	Albumin, globulins without IgG	8.4 ± 0.2	86.6 ± 3.1
Fr. 2	Wash	After affinity	12	Albumin, globulins without IgG	0.08 ± 0.1	2.5 ± 0.3
Fr. 3	Elution	After affinity and ultrafiltration	0.25	IgG_1, IgG_2, IgG_3, IgG_4	22.3 ± 3.7	14.3 ± 2.4

Supplemental Table S1. Analysis of fractions obtained during IgG antibodies preparation from 0.5 ml of serum. * calculated as the percentage of total protein content of sample before purification.

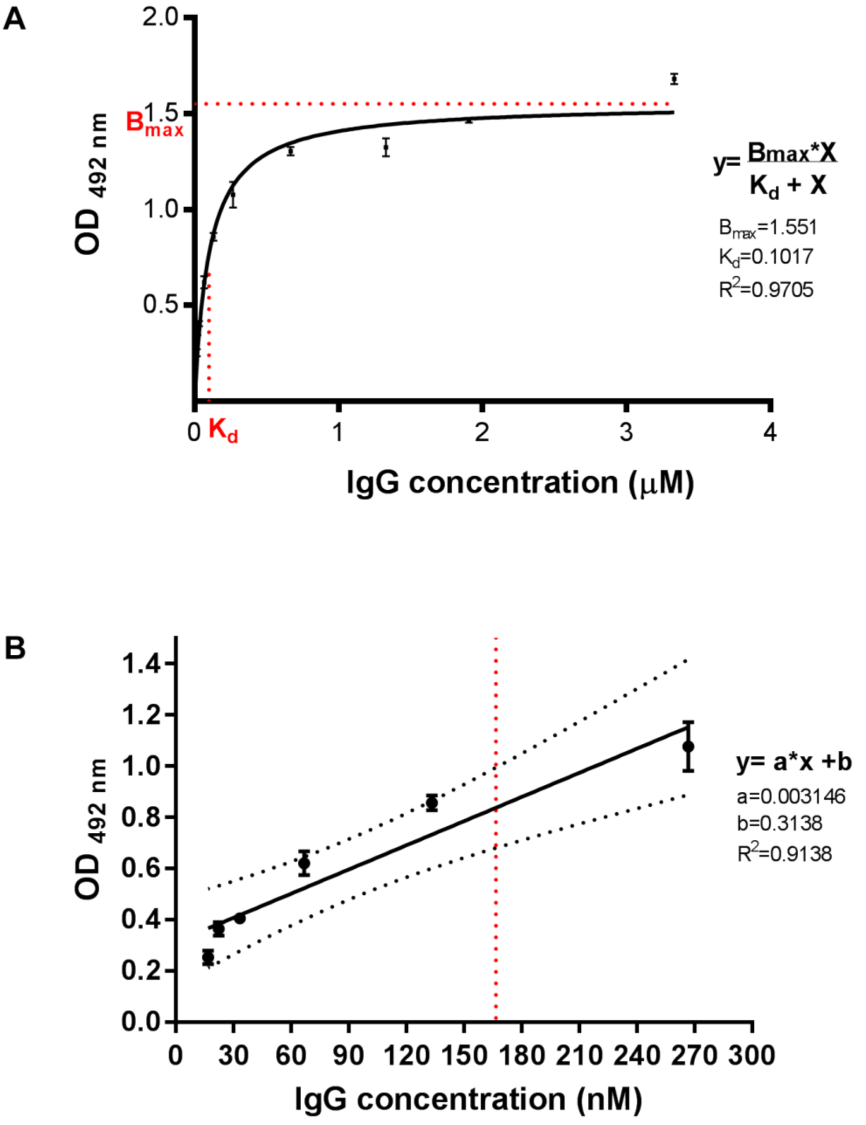

Supplemental Figure S1. ELISA dilution linearity test.

A) Equilirium assay. The binding strength of human IgG antibody to 1µg of C1P antigen was tested at ten different antibody concentration ranging from 0.0167 µM to 3.33 µM using an ELISA immunoassay. Kinetic parameters, i.e., equilibrium constant Kd and Bmax (indicated by red dotted lines), were determined by nonlinear regression (black curve) using a monovalent antibody model. **B)** A very good linear response (black line) reflecting binding of human IgG antibody to 1 µg of C1P antigen was obtained for the IgG antibody concentration range from 16.7 to 266.7 nM. 95% confidence interval is indicated by black dotted lines. The IgG concentration of 166.5 nM (marked in red dotted line) was chosen for further experiments.

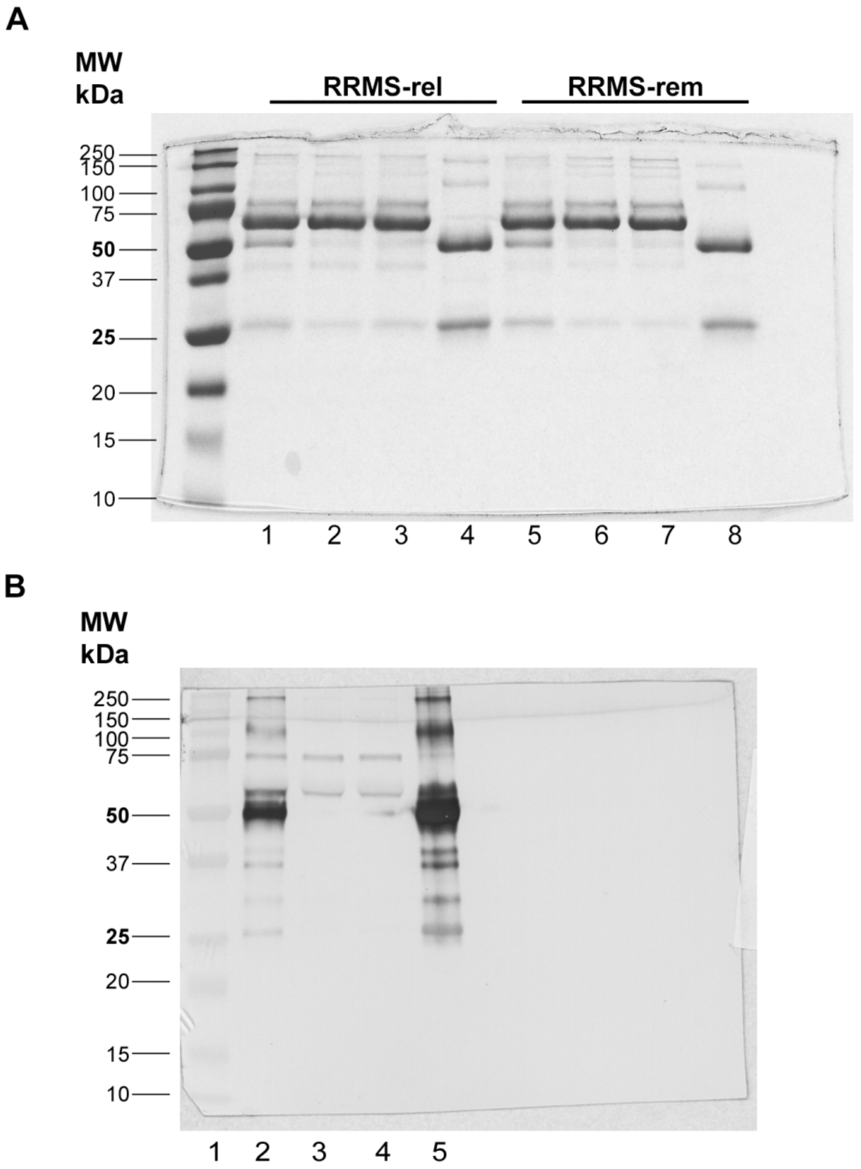

Supplemental Figure S2. The full-length gel **(A)** and blot **(B)** of the cropped images presented in the Fig. 1. The numbers indicate the same samples as shown in the main Fig. 1B and C, respectively.

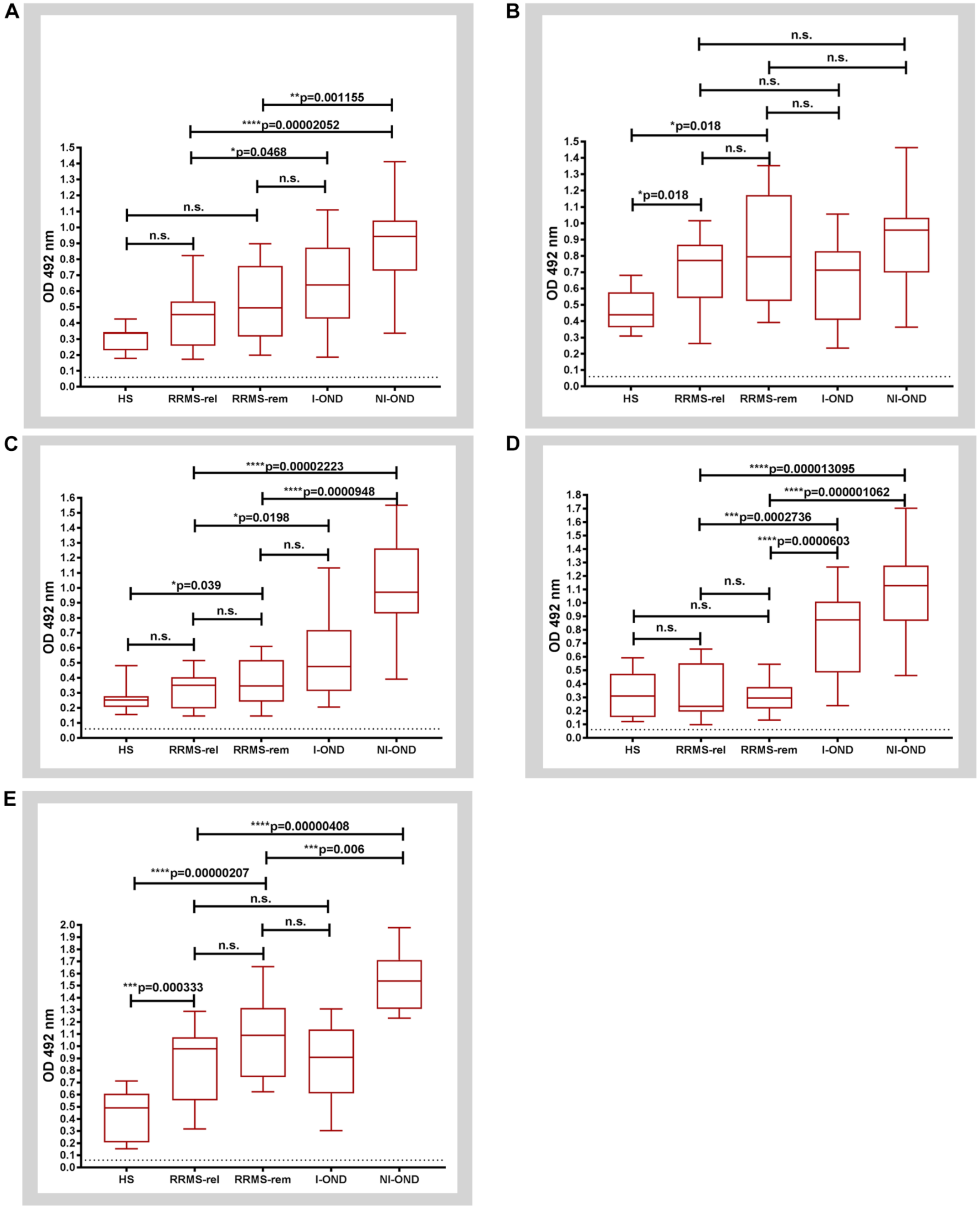

Supplemental Figure S3. Reactivity of IgGs purified from human serum against: **A)** C16:0-C1P, **B)** C18:0- C1P, **C)** C18:1-C1P, **D)** C24:0- C1P and **E)** C24:1- C1P by ELISA test. All boxes represent the 25th–75th percentile while horizontal lines inside of the boxes point out the median (50th percentile). Whiskers extend from the boxes indicate the range of the data - minimum and maximum values, accordingly. Depletion controls are indicated by dotted lines. Differences between groups were determined by the Mann- Whitney U test followed by Benjamini–Hochberg correction; adjusted p value is indicated.. HS (n=12), RRMS-rel (n=23), RRMS-rem (n=16), I-OND (n=13), NI-OND (n=13). Abbreviations: HS- healthy subjects; I-OND- inflammatory other neurological diseases; NI-OND- non-inflammatory other neurological diseases; n.s. –not significant; rel- relapse; rem- remission; RRMS- relapsing-remitting multiple sclerosis.

Group 1	Group 2	N1	N2	MEAN 1 ± SD	MEAN 2 ± SD	P value	Adjusted p value	Effect size	Magnitude
RRMS-rel	HS	23	12	0.4391 ± 0.1849	0.3085 ± 0.08119	0.042	0.0552857143	0.35	moderate
RRMS-rem	HS	16	26	0.5253 ± 0.2386	0.3085 ± 0.08119	0.043	0.0552857143	0.39	moderate
I-OND	HS	13	12	0.653 ± 0.28	0.3085 ± 0.08119	0.002	0.0045	0.63	large
NI-OND	HS	13	12	0.9145 ± 0.2757	0.3085 ± 0.08119	0.0001	0.00045	0.78	large
RRMS-rel	I-OND	23	13	0.4391 ± 0.1849	0.653 ± 0.28	0.026	0.0468	0.37	moderate
RRMS-rel	NI-OND	23	13	0.4391 ± 0.1849	0.9145 ± 0.2757	0.00000228	0.00002052	0.71	large
RRMS-rem	I-OND	16	13	0.5253 ± 0.2386	0.653 ± 0.28	0.228	0.251	0.23	small
RRMS-rem	NI-OND	16	13	0.5253 ± 0.2386	0.9145 ± 0.2757	0.000385	0.001155	0.63	large
RRMS-rem	RRMS-rel	16	23	0.5253 ± 0.2386	0.4391 ± 0.1849	0.251	0.251	0.19	small

Supplemental Table S2. The levels of reactivity of anti-C16:0-C1P serum IgG in patients with relapsing-remitting multiple sclerosis in relapse (RRMS-rel) and remission (RRMS-rel) phase, individuals with other neurological diseases with inflammatory (I-OND) or non-inflammatory (NI-OND) etiology as well as healthy subjects (HS). Pairwise comparisons were conducted using the Mann–Whitney U test with Benjamini–Hochberg correction. Abbreviations: C1P- ceramide-1-phosphate; SD- standard deviation.

Group 1	Group 2	N1	N2	MEAN 1 ± SD	MEAN 2 ± SD	P value	Adjusted p value	Effect size	Magnitude
RRMS-rel	HS	23	12	0.7011 ± 0.226	0.4714 ± 0.1202	0.006	0.018	0.46	moderate
RRMS-rem	HS	16	26	0.8232 ± 0.3282	0.4714 ± 0.1202	0.005	0.018	0.52	large
I-OND	HS	13	12	0.6457 ± 0.251	0.4714 ± 0.1202	0.068	0.1224	0.37	moderate
NI-OND	HS	13	12	0.9121 ± 0.2829	0.4714 ± 0.1202	0.00000535	0.0004815	0.74	large
RRMS-rel	I-OND	23	13	0.7011 ± 0.226	0.6457 ± 0.251	0.454	0.454	0.13	small
RRMS-rel	NI-OND	23	13	0.7011 ± 0.226	0.9121 ± 0.2829	0.034	0.0765	0.35	moderate
RRMS-rem	I-OND	16	13	0.8232 ± 0.3282	0.6457 ± 0.251	0.156	0.234	0.27	small
RRMS-rem	NI-OND	16	13	0.8232 ± 0.3282	0.9121 ± 0.2829	0.423	0.454	0.15	small
RRMS-rem	RRMS-rel	16	23	0.8232 ± 0.3282	0.7011 ± 0.226	0.275	0.353571428571429	0.18	small

Supplemental Table S3. The levels of reactivity of anti-C18:0-C1P serum IgG in patients with relapsing-remitting multiple sclerosis in relapse (RRMS-rel) and remission (RRMS-rel) phase, individuals with other neurological diseases with inflammatory (I-OND) or non-inflammatory (NI-OND) etiology as well as healthy subjects (HS). Pairwise comparisons were conducted using the Mann–Whitney U test with Benjamini–Hochberg correction. Abbreviations: C1P- ceramide-1-phosphate; SD- standard deviation.

Group 1	Group 2	N1	N2	MEAN 1 ± SD	MEAN 2 ± SD	P value	Adjusted p value	Effect size	Magnitude
RRMS-rel	HS	23	12	0.3264 ± 0.1111	0.2555 ± 0.08237	0.079	0.101571428571429	0.30	small
RRMS-rem	HS	16	26	0.3785 ± 0.1493	0.2555 ± 0.08237	0.026	0.039	0.42	moderate
I-OND	HS	13	12	0.5278 ± 0.2594	0.2555 ± 0.08237	0.002	0.0045	0.61	large
NI-OND	HS	13	12	1.022 ± 0.2998	0.2555 ± 0.08237	0.0000316	0.0000948	0.84	large
RRMS-rel	I-OND	23	13	0.3264 ± 0.1111	0.5278 ± 0.2594	0.011	0.0198	0.43	moderate
RRMS-rel	NI-OND	23	13	0.3264 ± 0.1111	1.022 ± 0.2998	0.00000247	0.00002223	0.79	large
RRMS-rem	I-OND	16	13	0.3785 ± 0.1493	0.5278 ± 0.2594	0.144	0.162	0.28	small
RRMS-rem	NI-OND	16	13	0.3785 ± 0.1493	1.022 ± 0.2998	0.0000231	0.0000948	0.79	large
RRMS-rem	RRMS-rel	16	23	0.3785 ± 0.1493	0.3264 ± 0.1111	0.272	0.272	0.18	small

Supplemental Table S4. The levels of reactivity of anti-C18:1-C1P serum IgG in patients with relapsing-remitting multiple sclerosis in relapse (RRMS-rel) and remission (RRMS-rel) phase, individuals with other neurological diseases with inflammatory (I-OND) or non-inflammatory (NI-OND) etiology as well as healthy subjects (HS). Pairwise comparisons were conducted using the Mann–Whitney U test with Benjamini–Hochberg correction. Abbreviations: C1P-ceramide-1-phosphate; SD- standard deviation.

Group 1	Group 2	N1	N2	MEAN 1 ± SD	MEAN 2 ± SD	P value	Adjusted p value	Effect size	Magnitude
RRMS-rel	HS	23	12	0.3278 ± 0.1865	0.3219 ± 0.1633	1.0	1.0	0	small
RRMS-rem	HS	16	26	0.3067 ± 0.108	0.3219 ± 0.1633	0.981	1.0	0.01	small
I-OND	HS	13	12	0.7883 ± 0.3106	0.3219 ± 0.1633	0.000451	0.0006765	0.66	large
NI-OND	HS	13	12	1.091 ± 0.319	0.3219 ± 0.1633	4.62e-06	1.386e-05	0.81	large
RRMS-rel	I-OND	23	13	0.3278 ± 0.1865	0.7883 ± 0.3106	0.000152	0.0002736	0.63	large
RRMS-rel	NI-OND	23	13	0.3278 ± 0.1865	1.091 ± 0.319	0.00000291	0.000013095	0.78	large
RRMS-rem	I-OND	16	13	0.3067 ± 0.108	0.7883 ± 0.3106	0.0000268	0.0000603	0.72	large
RRMS-rem	NI-OND	16	13	0.3067 ± 0.108	1.091 ± 0.319	0.000000118	0.000001062	0.83	large
RRMS-rem	RRMS-rel	16	23	0.3067 ± 0.108	0.3278 ± 0.1865	0.648	0.833142857142857	0.08	small

Supplemental Table S5. The levels of reactivity of anti-C24:0-C1P serum IgG in patients with relapsing-remitting multiple sclerosis in relapse (RRMS-rel) and remission (RRMS-rel) phase, individuals with other neurological diseases with inflammatory (I-OND) or non-inflammatory (NI-OND) etiology as well as healthy subjects (HS). Pairwise comparisons were conducted using the Mann–Whitney U test with Benjamini–Hochberg correction. Abbreviations: C1P-ceramide-1-phosphate; SD- standard deviation.

Group 1	Group 2	N1	N2	MEAN 1 ± SD	MEAN 2 ± SD	P value	Adjusted p value	Effect size	Magnitude
RRMS-rel	HS	23	12	0.869 ± 0.2784	0.4265 ± 0.2061	0.000148	0.000333	0.61	large
RRMS-rem	HS	16	26	1.073 ± 0.321	0.4265 ± 0.2061	0.00000046	0.00000207	0.82	large
I-OND	HS	13	12	0.8688 ± 0.314	0.4265 ± 0.2061	0.000756	0.0013608	0.64	large
NI-OND	HS	13	12	1.533 ± 0.2428	0.4265 ± 0.2061	0.000000385	0.00000207	0.85	large
RRMS-rel	I-OND	23	13	0.869 ± 0.2784	0.8688 ± 0.314	0.871	0.871	0.03	small
RRMS-rel	NI-OND	23	13	0.869 ± 0.2784	1.533 ± 0.2428	0.00000136	0.00000408	0.72	large
RRMS-rem	I-OND	16	13	1.073 ± 0.321	0.8688 ± 0.314	0.144	0.162	0.28	small
RRMS-rem	NI-OND	16	13	1.073 ± 0.321	1.533 ± 0.2428	0.004	0.006	0.52	large
RRMS-rem	RRMS-rel	16	23	1.073 ± 0.321	0.869 ± 0.2784	0.09	0.115714285714286	0.27	small

Supplemental Table S6. The levels of reactivity of anti-C24:1-C1P serum IgG in patients with relapsing-remitting multiple sclerosis in relapse (RRMS-rel) and remission (RRMS-rel) phase, individuals with other neurological diseases with inflammatory (I-OND) or non-inflammatory (NI-OND) etiology as well as healthy subjects (HS). Pairwise comparisons were conducted using the Mann–Whitney U test with Benjamini–Hochberg correction. Abbreviations: C1P- ceramide-1-phosphate; SD- standard deviation.

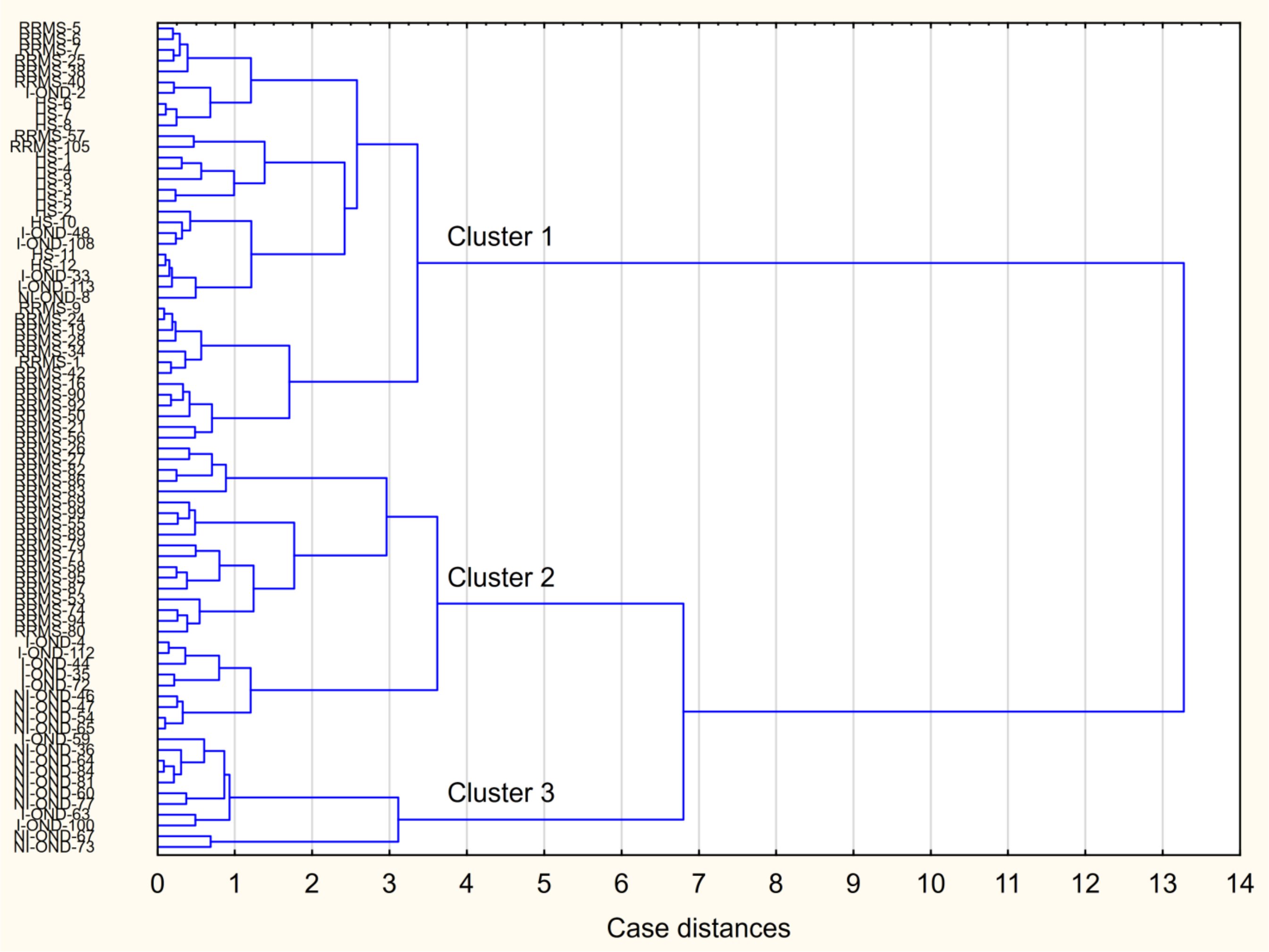

Group / Cluster No.	RRMS (n=39)	HS (n=12)	OND (n=26)
	The number of samples (percentage participation in whole group)		
3 (n=11)	0 RRMS (0%)	0 HS (0%)	11 OND (42.3%): 8 NI-OND (30.8%) and 3 I-OND (11.5%)
2 (n=27)	18 RRMS (46.2%): 10 RRMS-rel (25.6%) and 8 RRMS-rem (20.5%)	0 HS (0%)	9 OND (34.6%): 4 NI-OND (15.4%) and 5 I-OND (19.2%)
1 (n=39)	21 RRMS (53.8%): 13 RRMS-rel (33.3%) and 8 RRMS-rem (20.5%)	12 HS (100%)	6 OND (23.1%): 1 NI-OND (3.8%) and 5 I-OND (19.2%)

Supplemental Figure S4. Dendrogram of cluster analysis of serum IgG samples. The cluster analysis was performed for parameters which simultaneously complied the following criteria: they allowed for the differentiation of study groups and they had moderate or high clinical utility in the ROC curve analysis (AUC≥0.769). Each serum sample is represented by a vector of four parameters: anti-C16:0-C1P, anti-C18:0-C1P, anti-C24:0-C1P and anti-C24:1-C1P.

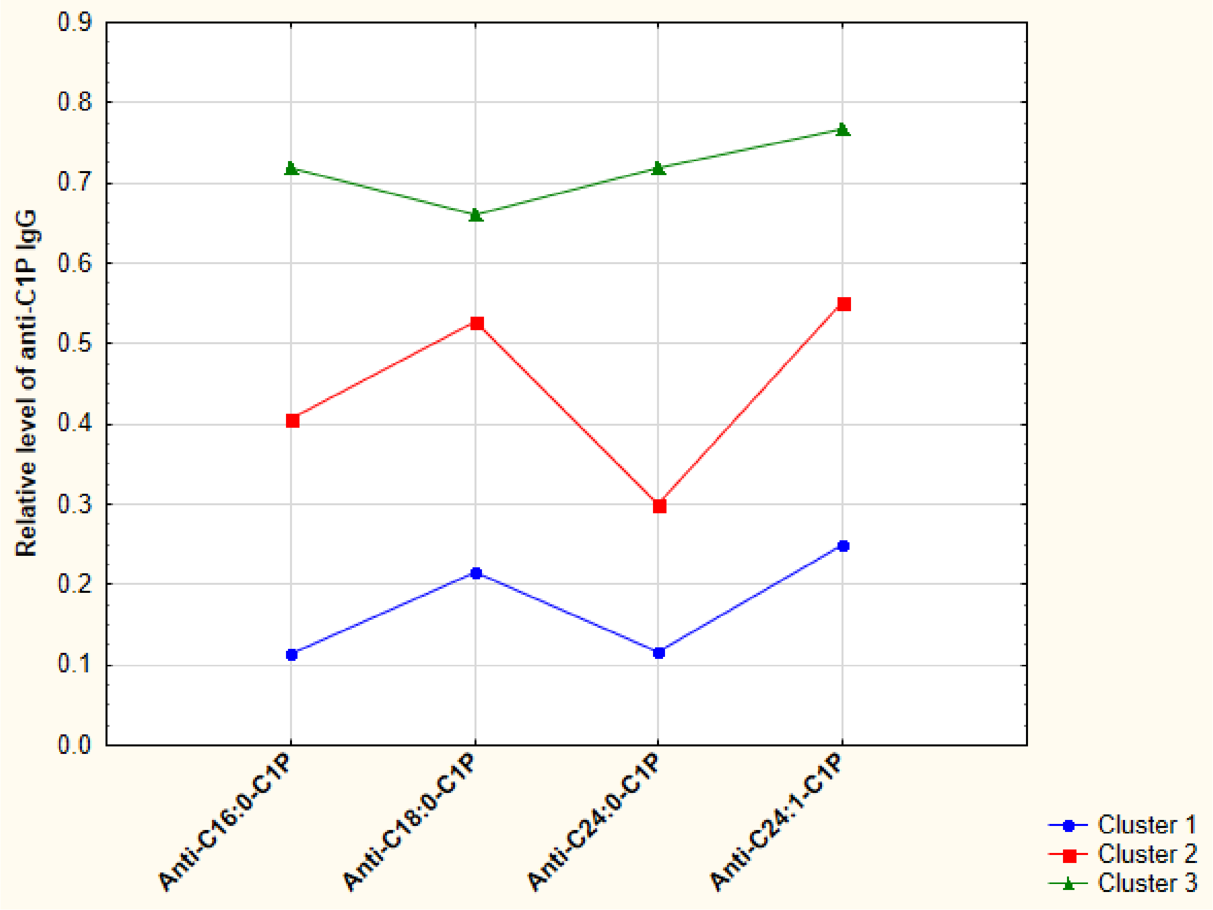

Group / Cluster No.	RRMS (n=39)	HS (n=12)	OND (n=26)
	The number of samples (percentage participation in each group)		
3 (n=11)	0 RRMS (0%)	0 HS (0%)	11 OND (42.3%): 8 NI-OND (30.8%) and 3 I-OND (11.5%)
2 (n=32)	23 RRMS (59%): 14 RRMS-rel (35,9%) and 9 RRMS-rem (23.1%))	0 HS (0%)	9 OND (34.6%): 4 NI-OND (15.4%) and 5 I-OND (19.2%)
1 (n=34)	16 RRMS (41.0%): 9 RRMS-rel (23.1%) and 7 RRMS-rem (17.9%)	12 HS (100%)	6 OND (23.1%): 1 NI-OND (3.8%) and 5 I-OND (19.2%)

Supplemental Figure S5. K-means clustering of serum IgG samples from RRMS, HS and OND groups. Four parameters: anti-C16:0-C1P, anti-C18:0-C1P, anti-C24:0-C1P and anti-C24:1-C1P IgG levels were taken for the analysis.

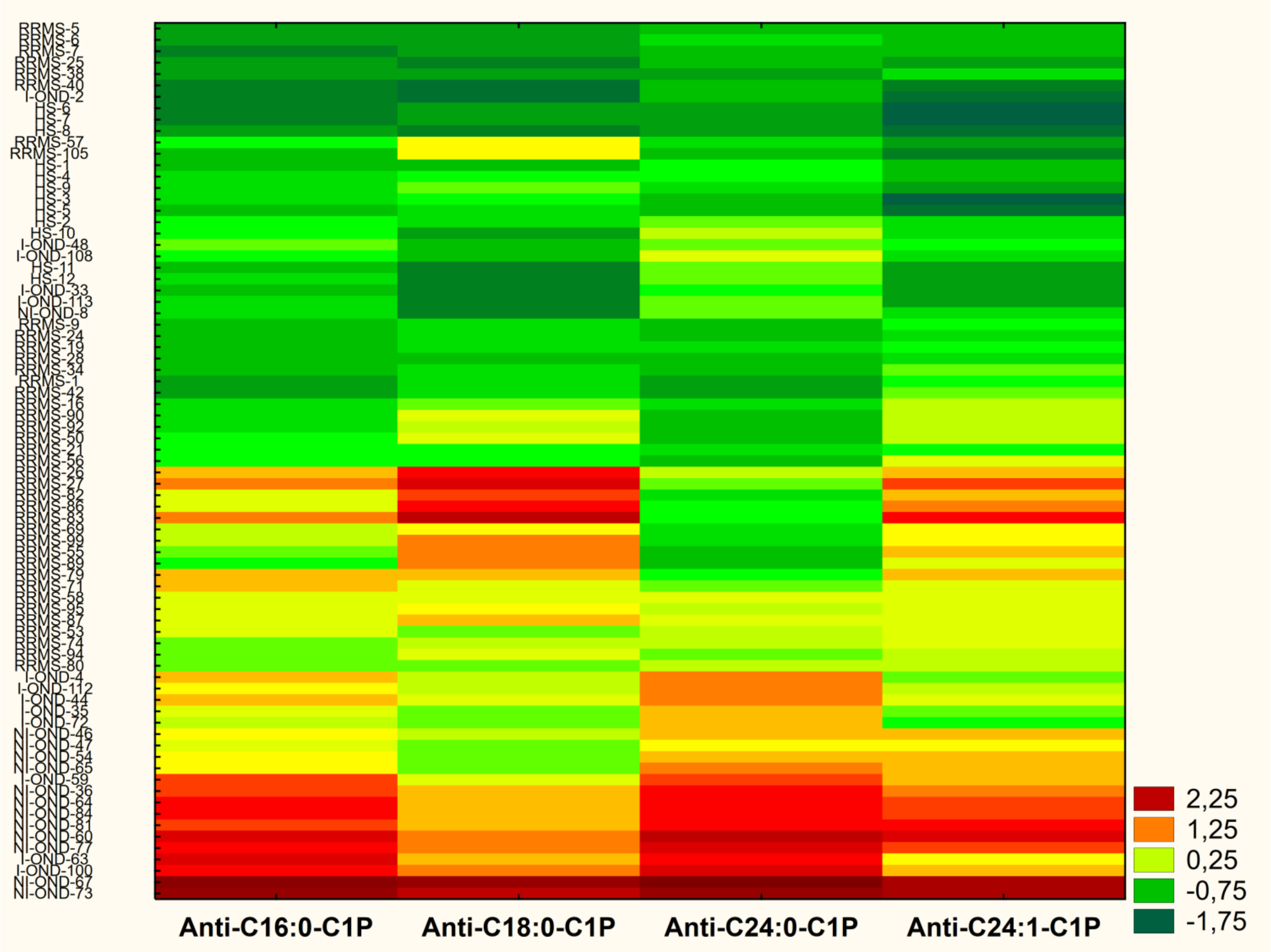

Supplemental Figure S6. Heat map showing distribution of selected anti-C1P IgG levels in the subjects examined.

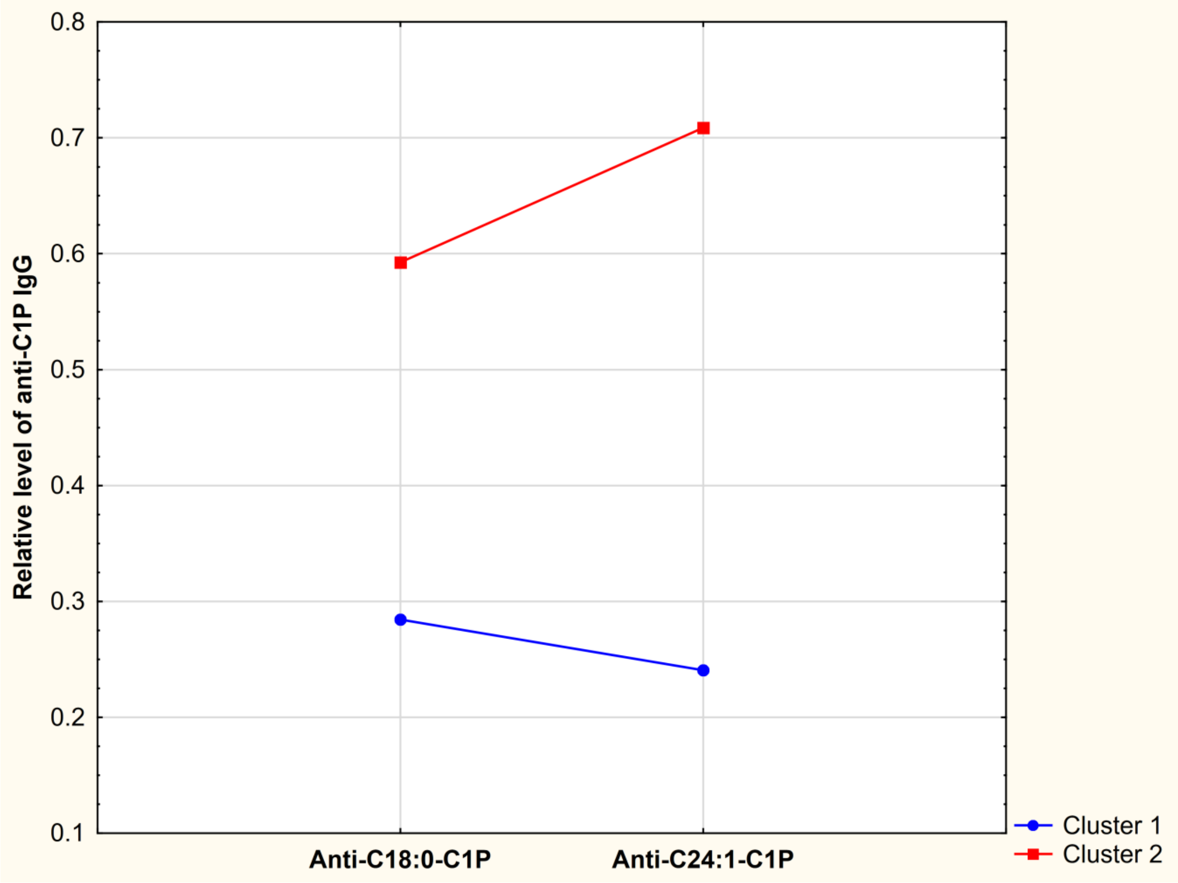

Group / Cluster No.	RRMS (n=39)	HS (n=12)
	The number of samples (percentage participation in whole group)	
2 (n=23)	23 RRMS (59.0%): 14 RRMS-rel (35.9%) and 9 RRMS-rem (23.1%)	0 HS (0%)
1 (n=28)	16 RRMS (41.0%): 9 RRMS-rel (23.1%) and 7 RRMS-rem (17.9%)	12 HS (100%)

Supplemental Figure S7. K-means clustering of serum IgG samples from RRMS and HS groups. Two parameters: anti-C18:0-C1P and anti-C24:1-C1P IgG levels were taken for the analysis.

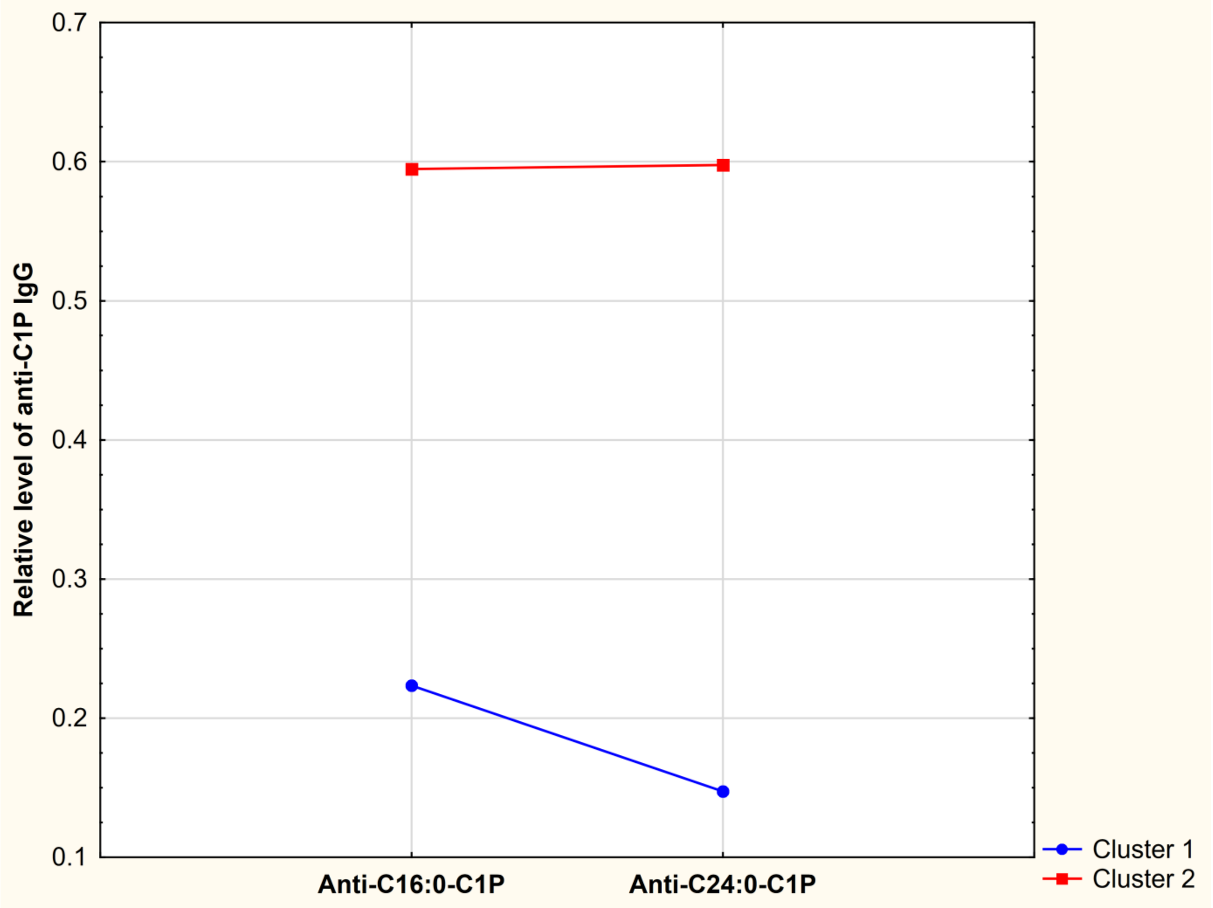

Cluster No. \ Group	**RRMS** (n=39)	**OND** (n=26)
	The number of samples (percentage participation in whole group)	
2 (n=20)	0 RRMS (0%)	20 OND (76.9%): 12 NI-OND (46.1%) and 8 I-OND (30.8%)
1 (n=45)	39 RRMS (100%): 23 RRMS-rel (59%) and 16 RRMS-rem (41%)	6 OND (23.1%): 1 NI-OND (3.8%) and 5 I-OND (19.2%)

Supplemental Figure S8. K-means clustering of serum IgG samples from RRMS and OND groups. Two parameters: anti-C16:0-C1P and anti-C24:0-C1P IgG levels were taken for the analysis.

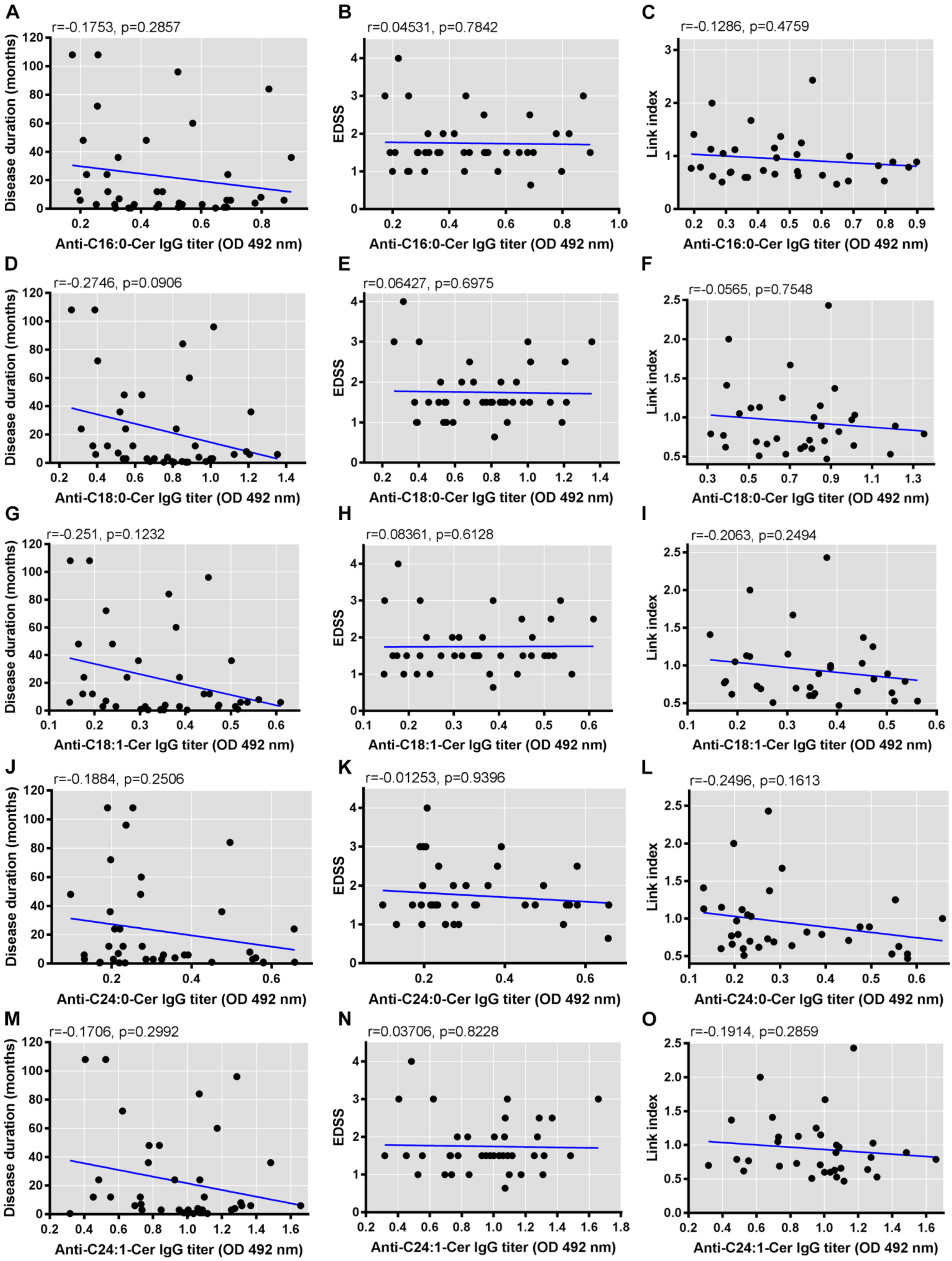

Supplemental Figure S9. Correlation of serum anti-C16:0-C1P **(panels A-C)**, anti-C18:0-C1P **(panels D-F)**, anti-C18:1-C1P **(panels G-J)**, anti-C24:0-C1P **(panels J-L)** and anti-C24:1-C1P **(panels M-O)** IgG derived from patients with RRMS with clinical parameters. Clinical variables included: disease duration **(panels A, D, G, J and M)**, EDSS **(panels B, E, H, K and N)** and Link index **(panels C, F, I, L and O)**. Statistical significance (p value) with appropriate Spearman's correlation coefficient predictor (r value) was provided. Abbreviations: C1P-ceramide-1-phosphate; EDSS- Expanded Disability Status Scale; RRMS- relapsing-remitting multiple sclerosis.
